# Reconciling models of interfacial state kinetics and device performance in organic solar cells: impact of the energy offsets on the power conversion efficiency†

**DOI:** 10.1039/d1ee02788c

**Published:** 2022-02-07

**Authors:** Mohammed Azzouzi, Nathaniel P. Gallop, Flurin Eisner, Jun Yan, Xijia Zheng, Hyojung Cha, Qiao He, Zhuping Fei, Martin Heeney, Artem A. Bakulin, Jenny Nelson

**Affiliations:** Department of Physics and Centre for Plastic Electronics, Imperial College London London SW7 2AZ UK Mohammed.azzouzi15@imperial.ac.uk; Department of Chemistry and Centre for Processable Electronics, Imperial College London London W12 0BZ UK jenny.nelson@imperial.ac.uk; Department of Hydrogen & Renewable Energy, Kyungpook National University Daegu 41566 Republic of Korea; Institute of Molecular Plus, Tianjin Key Laboratory of Molecular Optoelectronic Science, Tianjin University Tianjin 300072 P. R. China

## Abstract

Achieving the simultaneous increases in the open circuit voltage (*V*_oc_), short circuit current (*J*_sc_) and fill factor (FF) necessary to further increase the power conversion efficiency (PCE) of organic photovoltaics (OPV) requires a unified understanding of how molecular and device parameters affect all three characteristics. In this contribution, we introduce a framework that for the first time combines different models that have been used separately to describe the different steps of the charge generation and collection processes in OPV devices: a semi-classical rate model for charge recombination processes in OPV devices, zero-dimensional kinetic models for the photogeneration process and exciton dissociation and one-dimensional semiconductor device models. Using this unified multi-scale model in conjunction with experimental techniques (time-resolved absorption spectroscopy, steady-state and transient optoelectronic measurements) that probe the various steps involved in charge generation we can shed light on how the energy offsets in a series of polymer: non-fullerene devices affect the charge carrier generation, collection, and recombination properties of the devices. We find that changing the energy levels of the donor significantly affects not only the transition rates between local-exciton (LE) and charge-transfer (CT) states, but also significantly changes the transition rates between CT and charge-separated (CS) states, challenging the commonly accepted picture of charge generation and recombination. These results show that in order to obtain an accurate picture of charge generation in OPV devices, a variety of different experimental techniques under different conditions in conjunction with a comprehensive model of processes occurring at different time-scales are required.

## Abstract 2

We present a new framework to study organic photovoltaic devices in which a model that integrates device physics with excited state dynamics is applied to explain transient and steady-state spectroscopic and optoelectronic measurements.

## Graphical abstract

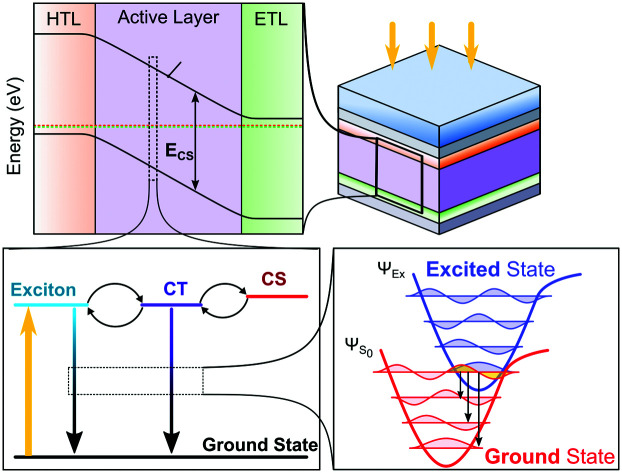

**Broader context**

Solution processable molecular semiconductors are attractive for low-cost, low-embodied-energy solar cells. The advent of novel acceptor molecules propelled the power-conversion efficiencies of these devices to over 18%. This efficiency improvement is attributed to efficient charge generation even at low offset between the donor and acceptor molecular energy levels. Kinetic interfacial models have been used to explain both the high photocurrent generation and low voltage losses in these devices but fail to explain the impact of the energy offsets on the device performance under operating conditions. In this work we combine an interfacial kinetic model with a device model and apply it to study devices with modulated energy offsets. Complementary measurements, namely, transient absorption spectroscopy, steady-state spectroscopy and transient optoelectronic measurements are used collectively to define the model parameters unambiguously. From this study, we find that when reducing the energy offset between donor and acceptor, not only is the gap between the lowest exciton and the charge-transfer (CT) state energy reduced, but, so is the gap between the CT and charge-separated (CS) states. This reduced CT-CS gap leads to poorer performance in low-offset devices due to accelerated back transfer of charges from the CS to CT state.

## 1. Introduction

Single junction organic photovoltaics (OPVs) have seen rapid development in recent years and have achieved remarkable power conversion efficiencies of over 18%.^1–4^ Many reports attribute this achievement to the realisation of efficient photocurrent generation in bulk-heterojunction (BHJ) blends with a small energy difference between the interfacial charge transfer (CT) state and the lowest single-component optical gap.^5^

Charge generation in organic solar cells is commonly accepted to proceed *via* the photogeneration of an exciton that is followed by its dissociation at the donor–acceptor heterointerface into a CT exciton which is finally dissociated into separate charges that deliver a photocurrent and which are denoted together by a charge-separated (CS) state.^6,7^ The charge generation efficiency thus depends on the difference in free energy between the lowest exciton (LE) state and the CT state or the CT and CS state,^8–10^ or more generally the free energy difference between lowest exciton and CS.^11^ Increasing these offsets comes at the expense of reducing the achievable open-circuit voltage (and therefore increasing voltage losses) for any given optical band gap. A long-standing goal has thus been establishing the minimum energy of these offsets that still allows for efficient charge generation.^12–17^ Charge generation efficiency in OPV devices has been shown to depend strongly on the competition between excited state recombination and dissociation.^18^ Reducing the offset between the exciton and the CT state compromises the charge generation efficiency since the driving force is reduced and the rate of exciton dissociation is reduced.^13,19^ This impact is reduced in blends with a long lowest exciton lifetime, which explains the high charge generation efficiency in some low offset blends.^9^ On the other hand, Karuthedath *et al.* recently showed that the yield of charge generation is rather strongly related to the ionisation energy offset between the donor and the acceptor.^20^ Kinetic zero-dimensional (0D) models are often used to quantify the impact of changing the energy offsets or the rates of dissociation and recombination on the device performances.^9,13,20,21^ Although the 0D kinetic models can explain to a certain extent the change in the charge generation efficiency and the voltage losses in the devices, even incorporating the equilibria between different state populations at open circuit,^21^ they do not account for the competition between the charge recombination and the charge extraction in the devices under operating conditions. Therefore, they could not explain the multiple reports of reduced fill factor (FF) in marginal offset bulk-heterojunction devices.^12–16,22^ As a compromise between the kinetic models and device models, Giebink *et al.* introduced an adapted diode equation that accounts for the properties of the interfacial state. They used this method to study the case of planar heterojunction solar cells and assess the impact of interfacial state properties on the device characteristics.^23^ For Bulk-heterojunctions, one-dimensional drift-diffusion device models have been extensively used to study the impact of the charge carrier transport and recombination properties on the device performance.^24,25^ Häusermann *et al.* previously introduced a coupled optoelectronic device model that considers the impact of the CT state dissociation and reformation.^26^ However, a device model that considers the different properties of the excited states in OPV devices and the different processes leading to charge generation or recombination is still lacking.

In this work, we introduce a framework that combines models that have been applied separately to describe different steps of the charge generation and collection processes in OPV devices: namely, a semi-classical rate model for charge recombination processes in OPV devices,^6,27,28^ zero-dimensional kinetic models for photogeneration process and exciton dissociation^13,29^ and one-dimensional semiconductor device models.^30,31^ Such a model clearly requires a large number of parameters that need to be consistently determined, and this leads to difficulties both in finding the correct combination of parameters and in providing insight into which physical or chemical parameter controls which device characteristic. Therefore, we also propose a methodology whereby key parameters for the series can be set sequentially by analysing different experimental measurements that are sensitive to different properties of the materials. Our methodology sacrifices perfect fitting of experimental data in order to produce a best minimum-parameter model that can explain the experimental data. With this approach, we can accurately evaluate the impact of changing a particular property of the material on the device performances with a limited parameter set. This approach enables consistent simulation of several experimental measurements (transient and steady state spectroscopy measurement, transient and steady-state optoelectronic measurement), while ensuring that the choice of parameters for the system are reliable.

We apply the developed model to investigate the differences in the charge generation, collection, and recombination process in OPV devices where the energy offset between the ionisation potential (or the Highest Occupied Molecular Orbital (HOMO) energy) of the donor and acceptor is reduced by fluorinating the donor. We investigate the origin of the reduced FF and *J*_sc_ of the lowest LE to CT energy offset blend by characterising the evolution of the excited species using steady state spectroscopy, transient opto-electronic measurements and ultrafast transient absorption (TA) techniques. If we assume that only the energy offset and three rate constants (for CT dissociation, reformation, and LE dissociation) are changing along the series of devices, we can reproduce all the different experimental results. These results suggest that by reducing the energy offset (in this case by fluorination of the donor) for the initial charge separation process, we not only reduce the rate of LE state dissociation but also reduce the CT dissociation rate constant and increase the back-formation of CT states from free charge carriers. These results help to rationalise the trade-off between increased *V*_oc_ and reduced FF and *J*_sc_ in marginal-offset blend. Using the results of the case study we then explore the correlation between changing the HOMO energy of the donor and the free energy offsets between either the LE and CT states, or the CT to CS states. Interestingly, we find that increasing the ionisation potential of the donor not only reduces the energy offset between the LE and the CT state, but it also reduces the free energy offset between the CT and CS state. We find that using the high temperature limit of the electron transfer rate constant established by Marcus we can fit the dependence of the dissociation rate constants on the two free energy offsets. We also find that the rate of back transfer from the free charges to the CT state to be strongly dependent on the free energy difference between the CS and CT state.

## 2. Theory

**Fig. 1:**
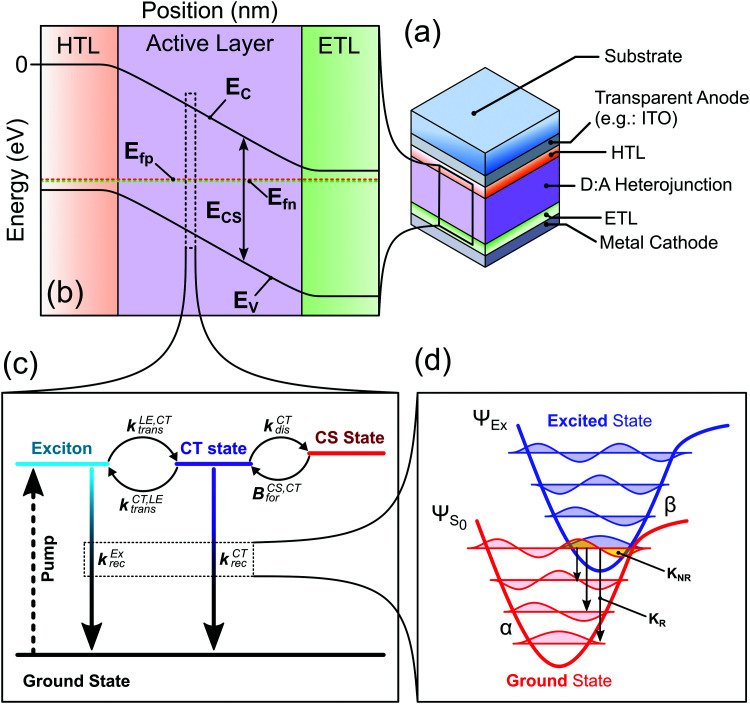
Device model considered in the paper. (a) Representation of a bulk heterojunction organic solar cell device, representing the different layers of the device stack. HTL and ETL stand for hole transport layer and electron transport layer, respectively. (b) Energy level diagram representation of the device under equilibrium generated, where *E*_c_ and *E*_v_ are the conduction and valence band energies, respectively; *E*_fp_ and *E*_fn_ are the hole and electron quasi-Fermi levels, respectively. The difference in energy (*E*_cs_ = *E*_c_ − *E*_V_) is the electric band gap. (c) Kinetic representation of the generation and recombination processes at each position of the active layer. The generation of the exciton is represented by the dotted arrow, more details about the other processes in the main text. (d) The potential energies of the ground state and an excited state as a function of reaction coordinate. The quantized vibrational modes are shown as the coloured waves, red for modes of the electronic ground state, and blue for modes of the excited state. The yellow overlap between the lowest vibrational state of the excited state and a vibrationally excited ground state indicates the non-radiative recombination pathway. The arrows depict possible radiative decay pathway. In the model, the recombination and absorption from the excited states (*i.e.*, the CT and lowest exciton) are described using this representation.

In this study we introduce a framework that combines different established models that were separately used to describe different aspects of the charge generation, recombination, and transport processes. The model as presented in this work is limited in the number of processes it considers since we aim to simplify the system as far as possible while still being able to explain the main physical processes pertaining to the charge generation in OPV devices (Fig. 1). Namely we consider the impact of the LE and CT state properties on the absorption and emission features of the device; the forward and backward transfer rates between the LE state, CT state and free charge carriers and their impact on the charge generation and recombination processes; and the impact of the charge carrier transport properties and spatial distribution on the device characteristics. Other physical processes could readily be included: such as (1) multiple trap states in the active layer and their impact on the charge carrier recombination and transport;^32^ (2) field dependent CT and LE state dissociation, by adding a field dependent factor to the dissociation rate constant as done by Peterson *et al.*;^29^ and (3) realistic exciton generation profile, by considering the optical properties of the different films and using a transfer matrix approach.^33^ Such options would increase the number of parameters needed and are not considered in this study.

The device is represented by a p-type/intrinsic/n-type (p–i–n) architecture (see Fig. 1a and b), where the p-type can be considered as the hole transport layer and the n-type the electron transport layer. The charge carrier (electron and hole) transport and the electric field in the device are described by the drift-diffusion equations.^34^ The active layer representing the bulk heterojunction is an effective medium where the donor and acceptor are well mixed, meaning that the sites at which lowest exciton (LE) and CT states can localise are evenly distributed in the layer. The charge generation and recombination processes in the active layer are assumed to occur *via* the LE and CT states, following the the kinetic model in Fig. 1c. This model for the charge carrier generation and recombination dynamics follows current understanding of those processes in organic solar cells.^35,36^ In this model we do not consider the LE and CT state to be mobile species.

The recombination and absorption properties of the excited states can be described in the framework of a semi-classical two-state model^6,37^ (Fig. 1d). In this model we mainly consider the lowest energy local exciton (LE) state and the lowest energy CT state. We estimate the rates of radiative and non-radiative recombination from the excited states using the Marcus-Levich-Jortner formula for the rate constants as described in.^12,28^ The radiative and non-radiative recombination rate constants are calculated based on properties of the excited-state-to-ground-state transition such as its free energy, oscillator strength of the transition and high and low frequency reorganisation energies. The absorption coefficient of the film can be approximated using the absorption rate constants of the CT and LE states and a contribution from higher energy states that follows the square root law of direct semiconductors with a band gap equal to the free energy of the LE state (more details can be found in ref. 28). Using the calculated properties of the states (emission spectra, radiative and non-radiative recombination rate constants) and the absorption coefficient of the film, we can estimate other important device characteristics (such as the radiative dark saturation current (*J*_0,rad_) and the radiative voltage limit (*V*_oc,rad_)), which are summarised in Table S1 (ESI†). Using *J*_0,rad_ we can calculate the population density at equilibrium of both the CT ([CT]_0_) and lowest exciton state ([LE]_0_) considering that the recombination of the photoexcited species only occurs through the CT or LE states (more details in Section 2 of the ESI†).

The different steps pertaining to the generation and recombination of the free charge carriers are represented in Fig. 1c. Following the generation of an exciton (volumetric rate *G*), the exciton either recombines with a first-order rate constant (*k*^LE^_rec_) or transfers to the CT state at the interface with the donor with a rate constant (*k*^LE,CT^_dis_). In this model, since we represent the active layer as an effective medium of mixed donor and acceptor phases, the diffusion of the lowest exciton from a pure donor or acceptor domain to a donor:acceptor interface is not effectively taken into account. The impact of the exciton diffusion on the average rate of exciton dissociation can however be represented by changing the exciton dissociation rate constant. The local density of lowest exciton (LE) states follows the continuity equation at any point in space:1
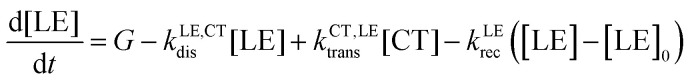
where *k*^CT,LE^_trans_ is the back-transfer rate constant from the CT state to exciton, [CT] is the density of CT states. The rate constants *k*^LE,CT^_trans_ and *k*^CT,Ex^_trans_ here are related to the population density at equilibrium of both the CT ([CT]_0_) and lowest exciton state ([LE]_0_). The rates and the equilibrium densities are considered to follow 
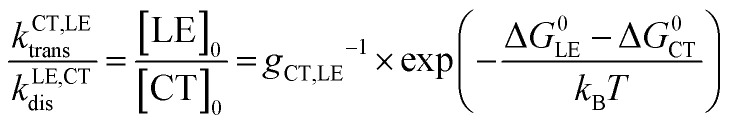
 to ensure detailed balance,^9^ where Δ*G*^0^_LE_ and Δ*G*^0^_CT_ are the free energies of the transition from the ground state to the LE and CT state, respectively, and *g*_CT,LE_ is the degeneracy ratio of CT to LE states. *G* is the local generation rate of lowest excitons, and can be modelled by any generation profile. In this work, we focus on the case where the generation is uniform in the active layer and the average generation rate (*G*_av_) under AM1.5 irradiance can be related to the absorptance of the film (*A*(*ħω*) as estimated using the approximation to the absorption coefficient in the previous paragraph) through:2
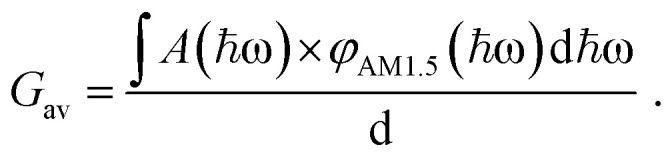
where *φ*_AM1.5_(*ħω*) is the spectral photon flux of an AM1.5 spectra. The CT state can also either recombine or form free charges (hole (p) and electron (n)). The local CT state population density ([CT]) follows the continuity equation3
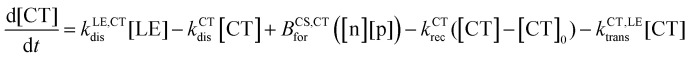
where *k*^CT^_dis_ is the rate constant of dissociation of CT state to free charges, *B*^CS/CT^_for_ the rate constant of formation of a CT state from free charge carriers and *k*^CT^_rec_ the rate constant for CT state recombination, [n] and [p] are the local density of electrons and holes, respectively. [CT]_0_ is related to the intrinsic charge carrier (*n*_i_) density in the semiconductor through ([CT]_0_*k*^CT^_dis_ = *B*^CS,CT^_for_*n*_i_^2^). The difference between the ionisation potential (*Φ*_IP_) and the electron affinity *Φ*_EA_, *i.e.*, the electric gap (*E*_CS_), can be related to other parameters through4
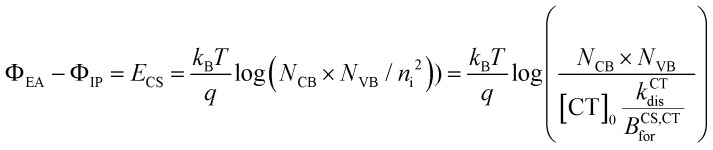
where *N*_CB_ and *N*_VB_ are the effective density of states in the conduction and valence bands, respectively. The recombination of the free charge carriers is therefore dominated by the formation of a CT state following the term *B*^CS,CT^_for_([n][p]). The continuity equations for the electron and hole densities can be expressed as5
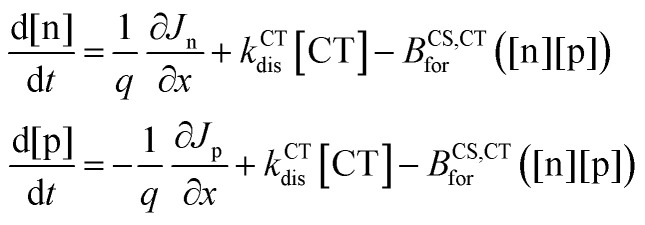
where *J*_n_ and *J*_p_ are the current density of electrons and holes in the layer, respectively. The Poisson equation is not affected by the CT and excitonic states as neither is charged. More details about the drift diffusion model and solver can be found in ref. 32 and 38.

In what follows, we use the model to simultaneously reproduce a set of experimental results of a series of devices with different energy offsets between the acceptor and donor LUMOs. The experimental measurements that can be explained simultaneously include steady state and transient luminescence and external quantum efficiency measurement, voltage losses measurement, ultra-fast transient absorption measurements and charge carrier recombination measurements (transient photo charge (TPQ) and charge extraction measurements). For this study we estimate the parameters of the model following the steps detailed in Diagram S1 in Section 2 of the ESI.† We first establish the free energy and reorganisation energies of the LE to ground state transition that best reproduce the steady state emission spectra and the exciton lifetime measured using transient photoluminescence on the pristine acceptor film (in our case the acceptor has the lower band gap of donor and acceptor). The LE to ground state properties are the same for the three devices studied here. Then we choose the free energy, reorganisation energies and oscillator strength of the CT to ground state transition for the different devices based on their electroluminescence, photoluminescence, and external quantum efficiency spectra as well as the measured voltage losses. In systems studied herein, we consider that the only property of the CT to ground state transition that changes along the series is its free energy. The ultrafast transient absorption measurements are then used to estimate the rates of CT and LE dissociation. Finally, measurements of charge carrier lifetimes in the operating device at 1 sun illumination are modelled to estimate the rate coefficient for CS to CT back formation (*B*^CS,CT^_for_).

## 3. Case study

### 3.1 System presentation

**Fig. 2:**
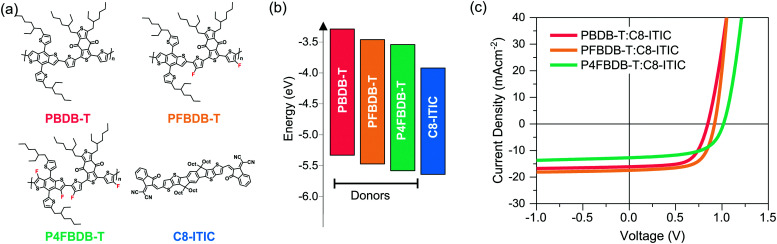
Materials and device performances. (a) chemical structure of the three donor polymers PBDB-T, PFBDB-T and P4FBDB-T and the acceptor C8-ITIC, (b) energy level (HOMO and LUMO) of the different donors and the acceptor, as estimated using cyclic voltammetry measurement according to results in ref. 12 and Table S4 (ESI†). (c) Current density voltage characteristics under simulated solar irradiation for the three devices considered in the study.

In this work we use the model to study devices based on poly[(2,6-(4,8-bis(5-(2-ethylhexyl)thiophene-2-yl)benzo[1,2-*b*:4,5-*b*′]dithiophene))-*alt*-(5,5-(1′,3′-di-2-thienyl-5′,7′-bis(2-ethylhexyl)benzo[1′,2′-*c*:4′,5′-*c*′]dithiophene-4,8-dione))], (PBDB-T) polymer derivatives as donors and C8-ITIC as an acceptor. Both the finding and the methodology presented herein is relevant to other type of bulk-heterojunction organic solar cells, and certainly to systems using oligomeric, low band gap acceptor molecules such as those used in most new high efficiency devices.^39^Fig. 2a displays the three donors: PBDB-T, the doubly fluorinated analogue PFBDB-T and the quadruply fluorinated P4FBDB-T, and the NFA acceptor, C8-ITIC, used in the study.^12,40^ As can be seen from the energy levels calculated from cyclic voltammetry measurements of thin films (ref. 12 and Table S4 in the ESI†), increasing the level fluorination of PBDB-T systematically reduces both the highest occupied molecular orbital (HOMO) and lowest occupied molecular orbital (LUMO) energies, without affecting the band gap. The energy offset between the HOMOs of the material, which affects the hole transfer process from the donor to the acceptor (C8-ITIC), thus decreases from more than 0.3 eV for PBDB-T:C8-ITIC to almost zero in P4FBDB-T:C8-ITIC, whilst the energy offset between the LUMOs of the two components of the blend, which affects the electron transfer process remains higher than 0.4 eV in all blends. It is important to note that all three polymers have similar absorption profiles, with absorption limited to wavelengths below 650 nm (Fig. S2, ESI†), whilst C8-ITIC absorbs strongly at longer wavelengths (up to 800 nm). This allows for selective excitation of the donor or the acceptor. Further, both GIWAXS^40^ and AFM^12^ measurements have shown that the morphological differences between non-fluorinated and fluorinated polymer:C8-ITIC blends are small, allowing us to ascribe differences in behaviour to the energetic differences described above.

Representative current density–voltage characteristics (*JV*) under simulated AM 1.5G irradiation for the three types of blend device considered in this study are presented in Fig. 2c, with the device performances reported in Table S5 (ESI†). All devices were fabricated in the so-called inverted solar cell architecture, ITO/ZnO/active layer/MoO_3_/Ag, and all were processed without additives (device fabrication details in the method section in the ESI†). In accordance with previous results, PFBDB-T:C8-ITIC shows the best performance, displaying a similar short-circuit current density (*J*_sc_) and fill factor (FF) to PBDB-T:C8-ITIC, but with an increased open circuit voltage *V*_oc_ which can be assigned to the decreased HOMO–HOMO offset described above. P4FBDB-T:C8-ITIC, on the other hand, displays the expected highest *V*_oc_, but significantly lower *J*_sc_ and FF compared to both other blends. It is important to note that similar reductions in those parameters have also been found when the HOMO–HOMO offset is reduced to close to zero in blends of P4FBDB-T with other acceptors such as ITIC and (21,40)bis[60]PCBM,^12^ and in many other donor–acceptor systems.^41,42^ The need to understand what limits the *J*_sc_ and FF of such low-offset blends is therefore of general importance.

### 3.2 Steady state absorption and emission measurements: determining the energies and lifetimes of the LE and CT excited-state-to-ground transitions

Within the framework introduced in the theory section, the properties of the excited states (the LE and CT states), control the absorption and emission properties of the devices. We therefore measured the electroluminescence, photoluminescence, and high-dynamic-range EQE of the three devices and aim to find the properties of the excited state that best reproduce these three measurements as well as the voltage losses of the three devices. First, since the acceptor (C8-ITIC) has a lower band gap than the polymers, we assume that the properties of the LE state are the same for the three blends and can be estimated by studying the pristine C8-ITIC films. In this work, we assume that the properties of the LE state do not change significantly between the blends and the pristine films.^43^ We use the steady state photoluminescence spectra and the transient photoluminescence dynamics of a pristine film of C8-ITIC to estimate the parameters of the LE state (mainly the free energy (Δ*G*^0^_LE_) and reorganisation energies of the transition from the ground state to the LE state). Table S6 (ESI†) summarises the values of these parameters chosen for the LE state to best reproduce both the measured exciton lifetime and the photoluminescence spectra (Fig. S3, ESI†).

**Fig. 3:**
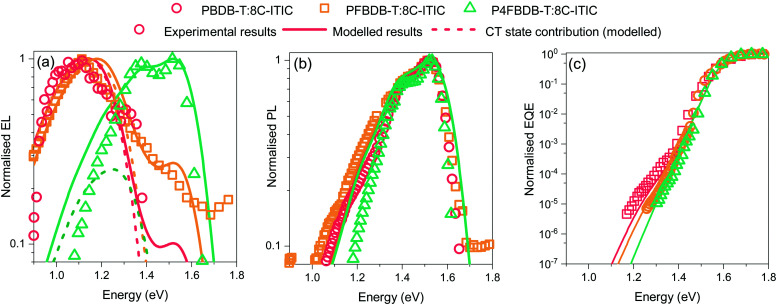
Steady state optical properties of the blends. (a) Normalised electroluminescence spectra of the three blend devices at an injection current of 100 mA cm^−2^. (b) Normalised Photoluminescence spectra of the devices. (c) Normalised EQE of the three blends. Solid lines show the model results.

Fig. 3 shows that upon fluorination of the donor, the electroluminescence spectrum blue shifts and the absorption edge (estimated using EQE spectra) gets sharper, whereas the PL spectrum remains fairly similar for the three blends and resembles the one from the pristine C8-ITIC (Fig. S4 in the ESI†). The difference between the EL and PL spectra is related to the nature of the injection of excited species (*i.e.* photo-generation of excitons in PL *versus* electrical injection of free electrons and holes in EL) with the effect that EL spectra reflect CT state emission more strongly than PL spectra. The shift in energy of the EQE edge and the EL indicate a blue shift of the CT state energy, as expected from the shift in the energy levels of the polymers (Fig. 2b). In the case of P4FBDBT, the electroluminescence and photoluminescence spectra strongly overlap. This indicates a strong contribution of the LE state to the electroluminescence spectra.

**Fig. 4:**
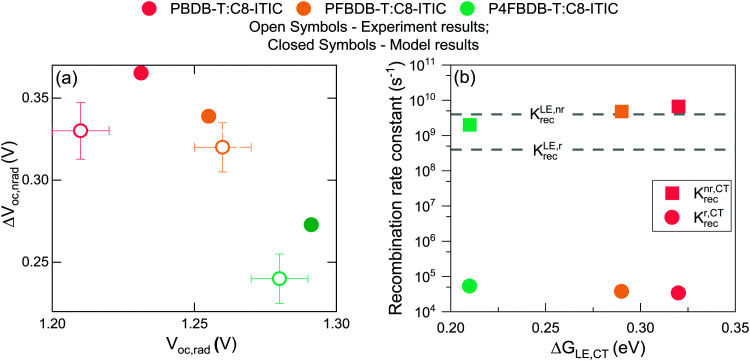
Voltage loss analysis. (a) Measured voltage losses for the three devices in closed circles and the results of the non-radiative voltage loss model in open circles. (b) Calculated rate constants of radiative and non-radiative recombination from the CT state for the three devices as a function of the free energy difference between the LE and CT state (the values of the free energies of the CT and LE state are the one in Table S7, ESI†).

We then determine the open-circuit voltage losses in the device from the measured external quantum efficiency and electroluminescence using the method introduced by Yao *et al.*^44^ The calculated radiative open circuit voltage (*V*_OC,rad_), and the non-radiative voltage losses (Δ*V*_oc,nr_) are presented in Fig. 4. Upon fluorination, *V*_OC,rad_ increases due to the sharpening of the EQE edge and the blue shift of the EL signal. The measured *V*_oc_ of the fluorinated polymer devices increases by more than the increase in *V*_OC,rad_ along the series of devices. This results in a significant decrease in Δ*V*_oc,nr_ for the P4FBDB-T:C8-ITIC blend relative to PBDB-T:C8-ITIC. These results are similar to the ones previously reported by Eisner *et al.* in ref. 12.

Using the model introduced we reproduce the absorption and emission properties of the devices as shown in Fig. 3, as well as their voltage losses in Fig. 4a, after applying the following constraints on the free parameters of the model:

1. The properties of the LE state (transition energy Δ*G*^0^_LE_, decay rate constant *k*^rec^_LE_, oscillator strength (Table S6 in the ESI†)) are the same for the three devices and in the pristine film (ESI,† Section 5).

2. The only property of the CT state that changes within the series of devices is its free energy of transition (Δ*G*^0^_CT_).

3. The oscillator strength of the CT state is four orders of magnitude lower than the LE state when the states are uncoupled, but benefits from a hybridisation effect related to the coupling between the two states and their free energy offset when offset is low.^12^

4. The rate constants of dissociation and reformation (*k*^LE,CT^_dis_, *k*^CT,CS^_dis_, *B*^CS,CT^_for_) are constrained to be consistent with the ones extracted by fitting the transient absorption (TAS) and transient photo-charge (TPQ) measurements (introduced later).

Considering the assumptions stated above, we can reproduce the experimental spectroscopic results (EQE, EL and PL) for the three blends studied (Fig. 3). The parameters of the CT state in the model for the three blends used to reproduce the experimental results are presented in Table S7 (ESI†). First, the shift in energy between the EL and the PL is well reproduced as a result of the different origin of the recombining excitons, *i.e.*, whether formed by electrically injected charges, tending to form CT states (case for the EL) or formed from optically generated excitons, that tend to be LE states (case for the PL).

The luminescence results obtained from the model for the three blends (Fig. 3) show that by just changing the energy of the CT state from 1.31 eV in the PBDB-T blend to 1.42 eV in the P4FBDB-T blend the contribution of the LE state to the EL is more pronounced. In the PBDB-T blend EL is dominated by the signal of the CT state, whereas for the P4FBDB-T blend, the contribution of the LE state to the EL is more pronounced. The contribution of the LE state to the EL spectrum is related to the population of the LE states that is occupied under a specific applied electric bias and the difference between the radiative rate constant of the LE and CT states. The radiative rate constant for the CT states (*k*^CT^_rec,r_, Fig. 4b), shows a small increase with decreased energy offset between the LE and the CT state due to the hybridisation effect, but it remains three to four orders of magnitude lower than *k*^LE^_rec,r_. This explains why, despite most of the recombination occurring through the CT state, the contribution of the LE state to the radiative recombination flux is not negligible, as also noted in other low offset bulk heterojunction solar cells.^9,21^

By only considering a change in the free energy of the CT state (Δ*G*^0^_CT_), we can accurately reproduce the voltage losses upon fluorination of the donor of the three devices (Fig. 4a). The increase in Δ*V*_oc,nr_ with reducing Δ*G*^0^_CT_ agrees with the energy gap law and the model proposed by Benduhn *et al.* for the voltage losses in OPV devices.^6^ The calculated overall recombination constant from the CT state (*k*^CT^_rec_, being the sum of the radiative and non-radiative rate constant, Fig. 4b) decreases as the free energy of the ground state to the CT state increases. In Fig. 4b, we present the results as a function the offset in free energy between the LE and CT (Δ*G*_LE,CT_ = Δ*G*^0^_LE_ − Δ*G*^0^_CT_) to show the impact of the free energy offset on the rates of recombination. Moreover, this decrease in rates of recombination suggests that the free charge carrier recombination flux should decrease upon fluorination, if the CT state decay were the rate-limiting component of this process. If all other device parameters were kept fixed, this would result in a clear improvement of the PCE upon fluorination of the device, as both the *V*_oc_ and the FF should increase due to a reduced recombination rate of the free charge carrier. In order to understand why the device performances do not follow the trend expected from these arguments, we investigate the dynamics of the photoexcited species further.

### 3.3 Early time dynamics: estimating the rates constants of LE and CT dissociation

**Fig. 5:**
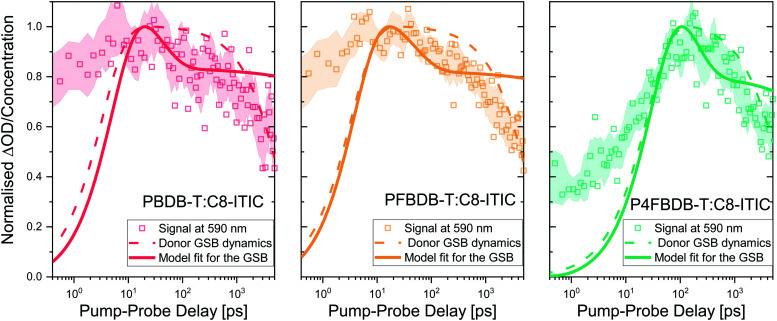
Hole transfer kinetics and kinetic model. Hole transfer kinetics and kinetic model for PBDB-T:C8-ITIC, PFBDB-T:C8-ITIC and P4FBDB-T:C8-ITIC, determined by monitoring the ground state bleach (GSB) of the donor. The optical signal at 590 nm is representative of a region where the donor ground state strongly absorbs. The dash dotted lines show the population of CT/CS states within the blend, as inferred from a global analysis of the transient absorption spectra (Section 9, ESI†). The solid lines show the results of the model for the GSB dynamics using the parameters in Table S8 in the ESI.†

We first probe the dynamics of the charge carrier generation at early times using femtosecond transient absorption spectroscopy in order to measure hole transfer kinetics from the NFA to polymer following the photoexcitation of the acceptor in films of each of the three polymer:NFA blends (Fig. 5). Previous studies have shown that energy transfer is likely to occur from donor to acceptor in these materials;^18,45^ to avoid this complication and simplify interpretation of the data, we choose a pump wavelength of 750 nm in order to selectively excite the acceptor species. The smaller bandgap of the acceptor material relative to all donor species precludes energy transfer from acceptor to donor; therefore, any appearance of the donor ground state bleach (GSB) can be assigned unambiguously to hole transfer.^46^

The evolution of the transient absorption signal following the photoexcitation of the acceptor of the three blends and the pristine C8-ITIC is presented in Fig. S6 (ESI†). The three blends exhibit bleach signals at *ca.* 650 nm which do not appear in the TA spectra of the pristine acceptor; consequently, we assign this feature to the GSB of the donor. As well as this, a second feature located at ∼750 nm is also apparent and can be attributed to the GSB of the acceptor through comparison with the steady-state absorption. We find that the TA spectra in our chosen wavelength region is well described by these two features alone, with no additional features apparent. The absorption of the donors and the acceptor in these blends (Fig. S2, ESI†) show a significant overlap even in the region where the donor absorption peaks. As a result, the transient absorption spectra of the three blends directly after the pulse (@ 0.2 ps) and after 4 ns both show a strong absorption feature in the region where the donors absorb (Fig. S7 and S2, ESI†). Using a global analysis algorithm considering two species (corresponding to the GSB of the donor and the acceptor; further details in the ESI,† Section 9) we were able to selectively track the dynamics related to hole transfer by monitoring the GSB of the donor. The dynamics obtained *via* this global analysis are given as dash-dot lines in Fig. 5. These show a slower rise than the TA data at 590 nm (where the donor strongly absorbs) due to the strong contribution of the acceptor absorption at that wavelength.

In the case of PBDB-T:C8-ITIC and PFBDB-T:C8-ITIC, a prompt rise in the donor GSB is observed, with a rise time on the order of a few picoseconds. In contrast, P4FBDB-T:C8-ITIC exhibits a rise time on the order of tens of picoseconds. We note that multiple previous studies have found similar slow charge generation processes in low-offset polymer:NFA blends.^18,45,47–52^ Given the high structural similarity between PBDB-T, PFBDB-T and P4FBDB-T and the conclusion of the GIWAXS^40^ and AFM^12^ measurements, it is reasonable to expect that the morphologies of the three blends are sufficiently similar so as to not significantly impact the rate of exciton migration, suggesting that these differences can be assigned to differing exciton dissociation rates (*k*^LE,CT^_dis_). The decay of the GSB after 100 ps is similar for the three blends suggesting no clear difference for the recombination processes in the three blends under the conditions of the transient optical experiments. The GA fits the decay of the GSB signal following the initial rise to a mono-exponential decay function, to roughly describe the trend in the raw experimental data.

Using the model presented earlier, we can simulate the GSB following photoexcitation of the acceptor LE state by simulating the evolution of the charge and exciton densities in the device following a short period of photoexcitation of the acceptor, representing the laser pulse while the device is kept at open circuit. The GSB signal is related to the population density of the CT state and the free charge carriers (electrons and holes). Modelling details are presented in the ESI,† Section 9.3. Since the device is kept in the dark during the TAS experiment, the density of free charges is small; consequently the rate of CT reformation, which is modelled as a second order process, is slow enough not to impact the dynamics of the GSB significantly on this fast time scale.^53,54^ With this assumption, we use the experimental GSB dynamics to estimate the dissociation rate constants of the LE and CT states (*k*^LE,CT^_dis_ and *k*^CT^_dis_, respectively). The modelled GSB dynamics are presented as solid lines in Fig. 5. The model accurately reproduces the rise of the GSB by considering a slower rate of dissociation of the LE state for the P4FBDB-T blend as compared to the other two (Table S8 for the rate constants, ESI†). The GSB dynamics from the model show a rapid decay that represents the recombination of the CT state before their dissociation, followed by a plateau that corresponds to the long-lived charge separated species (data in Fig. 5 after the initial peak). This is different from the first order decay that is shown by the GA dynamics fit.

By relaxing some of the assumptions made in the previous paragraph we could improve the fit of the model to the measured dynamics of the GSB for the blends, however, this would result in a worse reproduction of the device properties and the other experimental results. This is shown in the ESI,† Section 9.4 for the PFBDB-T:C8-ITIC blend. That case study emphasises the importance of considering a unique model that can describe different experimental measurements simultaneously.^55^ We note that the conditions of TA experiments relative to steady-state experiments (particularly with regards to excitation densities, which are typically far higher in TA experiments even when pump–pulse energies are weak) make it difficult to achieve a fit consistent with results gleaned from steady-state data.

The dissociation rate constants extracted from fitting the GSB dynamics show a significant difference between the large offset blend (PBDB-T:C8-ITIC) and the low offset on (P4FBDB-T:C8-ITIC) (Table 1). The CT dissociation rate constant (*k*^CT,CS^_dis_), decreases from 20 ns^−1^ for the PBDB-T blend to 4.1 ns^−1^ for the P4FBDB-T blend. For the P4FBDB-T blend, the dissociation of the CT state starts competing with the recombination rate constant from the CT state (*k*^CT^_rec_), which would impact the charge generation rate as well as the overall recombination of the free charge carriers. In the following sections, we will further assess the impact of changing these rate constants on the device performances by modelling the optoelectronic responses of the full device.

**Table 1:** Free input parameters that are modified along the device series to best reproduce the different experimental results. The difference in free energy between the CT and LE is calculated directly from the LE state energy and the free energy of the ground state to CT transition

Input parameter	Experimental measurement the parameter is extracted from:	PBDB-T	PFBDB-T	P4FBDB-T
Δ*G*^0^_CT_ (eV) [Δ*G*_LE,CT_]	Electroluminescence spectra (EL).	1.31 [0.32]	1.34 [0.29]	1.42 [0.21]
*k* ^CT,CS^_dis_ (s^−1^)	Donor ground state bleach (TAS)	20 × 10^9^	16 × 10^9^	4.1 × 10^9^
*k* ^LE,CT^_dis_ (s^−1^)	Donor ground state bleach (TAS)	170 × 10^9^	240 × 10^9^	25 × 10^9^
*B* ^CS,CT^_for_ (cm^3^ s^−1^)	Charge carrier lifetime (TPQ)	2 × 10^−11^	13 × 10^−11^	74 × 10^−11^

### 3.4 Charge carrier lifetime: estimating the rate coefficient of back transfer from the CS to the CT state

Using the absorption and emission spectra of the pristine acceptor and the blends, as well as the voltage losses of the three devices under 1 sun conditions, we have estimated properties of the LE and CT state that best reproduce these experimental results, specifically the free energies and reorganisation energies for the excited state to ground transition. Then from the GSB dynamics we have extracted the rate constants of CT and LE states dissociation that describe the three blends’ transient absorption spectra. Now to fully simulate the device under operational conditions where free charge densities are significant, we need to estimate the rate of CT reformation (*B*^CS,CT^_for_) and the free charge carrier mobilities and define the properties of the layers in the device structure (Table S14, ESI†).

For the device model, the properties of the ZnO and MoO3 transport layers, (*i.e.*, their energy levels, doping densities, and charge carrier mobilities) are chosen in a way that they should not affect the performance of the device. The dielectric constant of the blend layer and the effective density of conduction and valence bands (*N*_CB_ and *N*_VB_) are chosen to agree with reported values in the literature for similar materials.^24,56^ It is important here to note that the values of *N*_CB_ and *N*_VB_ would mainly impact the calculated energy of the electric band gap (*E*_CS_) but do not impact the intrinsic charge carrier density (*n*_i_) which is calculated based on the population density of CT states at equilibrium ([CT_0_]) nor the rates of dissociation and reformation (eqn (4) and Section 2 in the ESI†). In the limit where we model the device as a three-layer *p*–i–*n* dual homojunction model, the choice of *N*_CB_, *N*_VB_ within reasonable values, and consequently *E*_CS_, would not impact the predicted device performance. Moreover, the parameters that would strongly impact the device performance are the charge carrier mobilities (*μ*_e_, *μ*_p_) and the thickness of the devices. First, all three blends are considered to have an active layer thickness of 100 nm considering that the measured active-layer thickness of selected devices of the three blends were in the range [90 to 110 nm]. For this study, we use a free charge carrier mobility value of 3 × 10^−4^ cm^2^ s^−1^ V^−1^ for both species and for all three blends, which is consistent with values measured using space-charge-limited current (SCLC) for these blends (Section 10 in the ESI,† and ref. 40). We don’t distinguish between mobilities of different carriers or blends, despite some differences in the parameters obtained from SCLC analysis, because of the known difficulties in determining charge carrier mobility accurately in bulk heterojunctions, and because all the obtained values lie within the same order of magnitude (Section 10 in the ESI†). With this approach we can focus on the impact of changing the free energy offset on the dissociation and recombination process rather than the impact of the charge carrier transport (discussion in Section 11 of the ESI†).

For the reformation of CT state rate constant (*B*^CS,CT^_for_), we found analytically and by exploring the parameter space for the simulation (Section 11.1 in the ESI†) that the lifetime of the free charge carrier is mainly affected by the effective bimolecular recombination rate constant defined as 
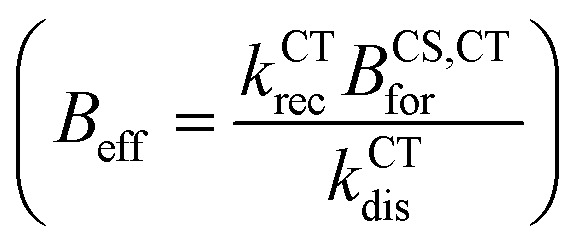
. Therefore, we aim to choose *B*^CS,CT^_for_, that best reproduces the effective charge carrier lifetime as measured using transient photo-charge (TPQ).^32^ The charge carrier lifetime measure using TPQ (*τ*_Q_) is more representative of pair recombination dynamics then the measured transient photovoltage lifetime since it avoids limitations due to charge carrier transport and capacity effects as explained in ref. 32 (detailed analysis of the results is presented in the ESI,† Section 11.2).

**Fig. 6:**
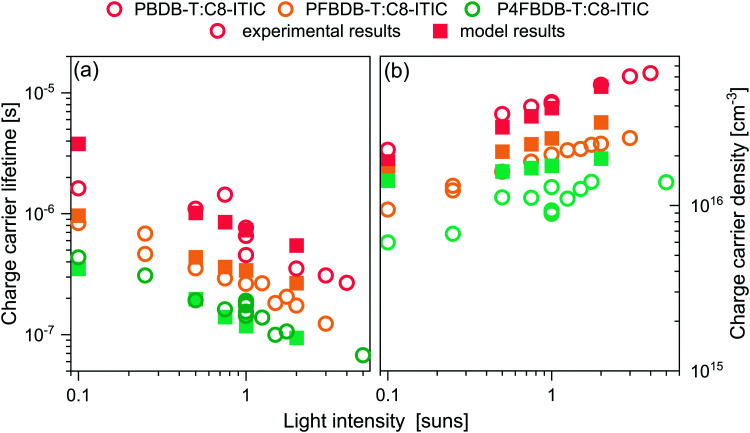
Experimental (open symbols) and calculated (filled symbols) charge carrier lifetime (a) and charge carrier density (b) at open circuit voltage and under different light intensities (represented as suns equivalent light intensity).

The charge carrier lifetimes, measured as a function of light intensity, are presented in Fig. 6a. The measured *τ*_Q_ decreases with decreasing energy offset for hole transfer and is the lowest for the P4FBDB-T:C8-ITIC device at all light intensities. Under 1 sun simulated illumination, the lifetime of the free charge carriers for the P4FBDB-T was around 0.1 μs which is an order of magnitude lower than *τ*_Q_ for the PBDB-T blend. The short lifetime of the free charge carrier points toward faster recombination of the photoexcited charges and therefore a higher *B*_eff_. We also measured the density of free charge carriers at different light intensities using charge extraction and found it to decrease with decreasing energy offset, at all light intensities.

The simulated charge carrier lifetime was estimated by fitting the decay of the excess charge carrier density introduced by a short laser pulse. This approach is similar to the way the charge carrier lifetime is measured experimentally using TPQ (more details about the simulation in Section 11.2 of the ESI,† and ref. 32). To reproduce these experimental results (Fig. 6), we use the parameters extracted from fitting the previous results (Table 1 summarises the only parameters changing between the three devices), as well as the fixed device parameters shown in Table S15 (ESI†), and allow *B*^CS,CT^_for_ to vary between blends to account for the change in the charge carrier lifetimes (Table 1). As shown in in Fig. 6, the results of the model for the three devices reproduces the experimental results for both the charge carrier lifetime and charge carrier densities.

### 3.5 Device performance

**Fig. 7:**
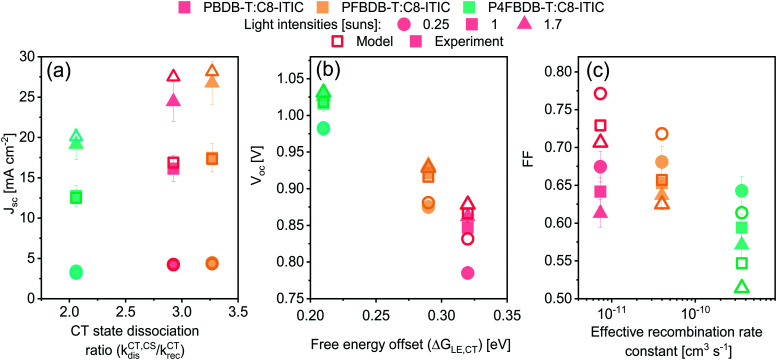
Measured (closed symbols) and simulated (open symbols) device performances of the three PBDB-T:C8-ITIC, PFBDB-T:C8-ITIC and P4FBDB-T:C8-ITIC devices, at three different light intensities, plotted against the parameter combination that most affects each of the main external solar cell parameter (*J*_sc_ (a), *V*_oc_ (b) and FF (c)).

All the parameters of the model for the three devices in the series studied have now been determined by reproducing the different experimental measurements introduced above, namely: (1) absorption and emission spectra; (2) voltage loss analysis; (3) GSB of the donor dynamics; (4) the lifetime and density of charge carriers at different light intensities. The fitted parameters are given in Table S6 (ESI†) for the LE properties, Table S7 (ESI†) for the CT state properties, Table S8 (ESI†) for the rate constants of the different processes and Table S15 (ESI†) for the device structure and transport properties. Most importantly, only four free parameters are assumed to change along the series (considering the similarities between the three blends), specifically: the energy of the CT state, the dissociation rate constant of then LE state and the CT state, and the reformation rate constant of the CT state. Using only these four parameters, we are able to accurately reproduce the short circuit current density (*J*_sc_) and open circuit voltage (*V*_oc_) for the three devices, as well as the trend in the FF along the series (Fig. S18, ESI†). Focusing on the four free parameters considered in Table 1, we identify which set or combination of parameters affects each one of the *J*_sc_, *V*_oc_ or FF the most (Fig. 7). We also explored the influence of these four free parameters as well as the charge carrier mobility on the device characteristics considering a larger parameter space, as discussed in the ESI,† Section 11.1. The analysis suggests that the drop in *J*_sc_ for the P4FBDB-T blend is primarily related to the ratio of the CT state dissociation rate constant to its recombination rate constant (*k*^CT,CS^_dis_/*k*^CT^_rec_). The impact of the reduced LE dissociation rate constant in the P4FBDB-T blend on the *J*_sc_, is lower than that of the reduced CT state dissociation rate *k*^CT,CS^_dis_, due to the lower ratio *k*^CT,CS^_dis_/*k*^CT^_rec_ compared to *k*^LE,CT^_dis_/*k*^LE^_rec_. In other words, the dissociation of the LE state, even if it is considerably reduced, is still efficient considering the slow LE recombination rate constant, whereas for the CT state its dissociation is reduced. As presented in the ESI,†*V*_oc_ is mostly impacted by the properties of the excited states (LE and CT state). Of these, the CT state Gibbs free energy (Δ*G*^CT^_0_) is the only parameter changing along the series and is represented in Fig. 7 by the change in the free energy offset between the LE and CT state. The FF is mainly affected by the change in the effective recombination rate constant 
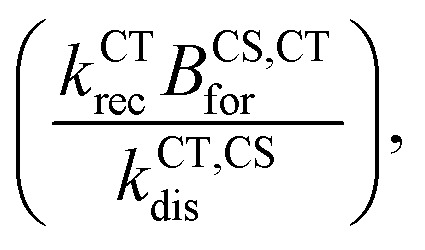
 considering that the charge carrier mobility is the same for the three blends in the series, as it affects the charge carrier lifetime discussed in Sections 5.4 and 11 in the ESI.† These observations agree with the larger parameter exploration presented in Section 11 of the ESI.†

## 4. Discussion

We have introduced in this paper a comprehensive model of OPV devices that can reproduce their optical properties, charge carrier dynamics, ultrafast charge generation processes and steady state device performances. We then applied the model to study a series of chemically similar bulk heterojunction devices with different energy offsets between the molecular orbitals of the donor and the acceptor. We have shown how the set of free parameters required by the model can be extracted by reproducing different sets of experimental results. It is important to emphasise that to extract a reliable set of parameters for the model we need to consider a variety of experimental measurements under different conditions. By only focusing on fitting or reproducing a smaller set of experimental measurement or fitting each experimental result separately, we risk using a set of parameters that does not accurately describe the system. For example, we demonstrated in Section 9.4 of the ESI,† that a parameter set can be used to accurately reproduce the EQE, EL spectra and hole transfer dynamics of the PFBDB-T:C8-ITIC blend. However, in this case, by overlooking the voltage losses results and the charge carrier lifetimes, this particular set of parameters would result in significantly diverging device performances.

**Fig. 8:**
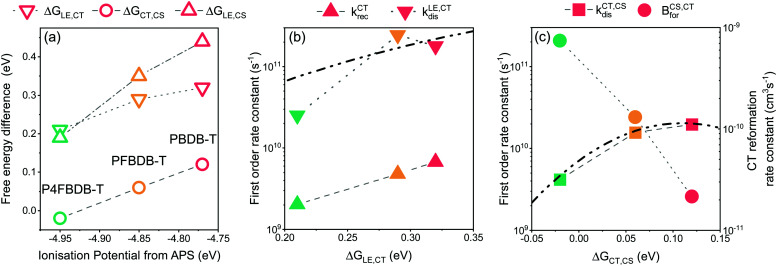
Impact of changing the ionisation energy of the polymer on the energy offsets and the different rates of dissociation and reformation. (a) Free energy offsets between the LE, CT and CS state as a function of the ionisation potential of the polymer. The Free energy offsets are taken from the parameters used in the model. (B) The rates of exciton dissociation and CT state recombination as a function of the LE to CT free energy offset. (C) The dissociation and reformation rate constants of the CT state as a function of the CT to CS free energy offset. The dot dashed lines in figure b and c are the fit to the dissociation rate constants using the Marcus electron transfer rate equation in the high temperature limit.

For the systems studied here, the free energy difference between the LE and CT state, Δ*G*_LE,CT_, has been explicitly considered as a free parameter of the model and was not explicitly related to a change in the energy levels of the donor. On the other hand, the impact of the fluorination of the donor on the free energy of the free charge carriers (*i.e.*, the electric gap energy *E*_CS_) is implicitly calculated based on eqn (4). The free energy of the CS state is related to the equilibrium between the CT and CS state (*i.e.*: the rate constant of the CT dissociation and reformation) and the effective density of states in the bands (*N*_CB_ and *N*_VB_). The energy of the CS state can be estimated from the difference between the ionisation potential (IP) of the donor and the electron affinity (EA) of the acceptor.^20^ Interestingly using the IP and EA values reported by Karuthedath *et al.*^20^ for PBDB-T-2F (which is structurally like PFBDB-T) and ITIC (which has similar electronic properties as C8-ITIC) we find *E*_cs_ = IP(D) − EA(A) = 1.25 eV, which is close to the value used in the model for the PFBDB-T:C8-ITIC blend (*E*_cs_ = 1.28 eV). Moreover, the change in E_CS_ considered in the model for the three blends is similar to change in both the measured IP using air photoelectron spectroscopy and the HOMO level estimated from cyclic voltammetry measurement (ref. 12, Fig. 8a and Fig. S19, ESI†). From the model results, we find that both free energy offsets, Δ*G*_LE,CT_ and Δ*G*_CT,CS_ (here defined as Δ*G*_CT,CS_ ≈ Δ*G*^0^_CT_ − *E*_CS_), reduce with reducing offset energy between the HOMO of the donor and the acceptor. This suggests that the difference in energy between the CS state and the CT state is affected by the energetics of the donor and acceptor molecules.^36^ Δ*G*_LE,CT_ in the P4FBDB-T blend is reduced by 110 meV relative to the PBDB-T blend, whereas Δ*G*_CT,CS_ is reduced by 140 meV. This shows that in our system the difference in free energy between the CT and the CS states is as affected by the change in the HOMO (or the ionisation energy) of the donor as the difference in energy between the LE and CT state. The impact of fluorinating the donor on the free energy offsets Δ*G*_LE,CT_ and Δ*G*_CT,CS_ is not yet clear. It may be related to an electrostatic offset on the energy level profile near the donor:acceptor interface due to the quadrupole moments of the molecules.^20^ Alternatively, the reduced Δ*G*_CT,CS_ gap may conceivably result from an increase in the binding energy of the CT state due to the hybridisation between the LE and CT state.^12,57^ We note that, whatever the underlying mechanism, the changes in free energy offset are not related in a trivial way to the polymer ionisation energy (as seen in Fig. 8a, especially for the low offset system).

The dissociation rate constants (*k*^CT,CS^_dis_ and *k*^LE,CT^_dis_) in this study are found to be strongly affected by the free energy offset between the states (Δ*G*_LE,CT_ and Δ*G*_CT,CS_). This agrees with previous reports for both the dissociation of the LE state and the CT state (ref. 8, 58 and 59 for the LE state dissociation, andref. 20 and 36 for the CT state dissociation). The impact of the free energy differences between the states and the rates of electron transfer can be modelled within the framework of the semiclassical electron transfer rate theory due to Marcus.^60^ Using the Marcus rate formula in the high temperature limit, we can estimate the rates of LE and CT dissociation considering the energy difference between the states (Δ*G*_LE,CT_ and Δ*G*_CT,CS_), and the electronic coupling between the states as well as the reorganisation energies related to the transition^61,62^ (Fig. 8b and c). For our system, we can reproduce the change in the dissociation rate constant by considering that the electronic coupling and reorganisation energies related to each dissociation process are the same across the series. For the LE dissociation rate constant we obtain good agreement between the Marcus rate constant and the *k*^LE,CT^_dis_ used in the model when we assume an electronic coupling between the two states of 30 meV and a reorganisation energy of 540 meV. (Fig. 8b and the ESI,† Section 13 for more information). For the CT state dissociation (*k*^CT,CS^_dis_), we find that a Marcus rate constant with a coupling of 4 meV and a reorganisation energy of 110 meV describes the correlation between the calculated Δ*G*_CT,CS_ and the chosen *k*^CT,CS^_dis_ (Fig. 8c). Using these results, *k*^CT,CS^_dis_ for the lowest offset blend (P4FBDB-T:C8-ITIC) can be increased by either improving the electronic coupling between the CT and CS state or reducing the reorganisation energy of the transition. Increasing *k*^CT,CS^_dis_ for the lowest offset blend could lead to a considerable increase in the PCE up to 13.4% (ESI,† Section 15).

The reformation rate constant of the CT state (*B*^CS,CT^_for_) is increased with decreased energy offset between the donor and the acceptor HOMO along the devices considered in this study. The rate constant *B*^CS,CT^_for_ has been previously described by an encounter probability in the Langevin recombination framework 
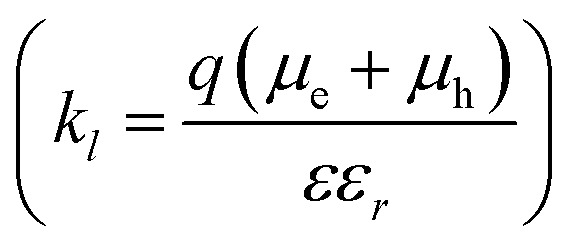
.^7,63^ To explain the trend in *B*^CS,CT^_for_ along the series using the Langevin encounter probability framework (*B*^CS,CT^_for_ is a function of the charge carrier mobilities), the average charge carrier mobility for the PBDB-T would have to be 40 times lower than that of the P4FBDB-T blend (ESI,† Section 14). This change in the charge carrier mobility does not agree with the estimated charge carrier mobilities measured using SCLC, and assuming such a low value for the charge carrier mobility would significantly impede the device performance of the PBDB-T blend. These findings imply that *B*^CS,CT^_for_ is not solely limited by an encounter probability of the free charge carries, rather it appears to be related to the energetics at the donor and acceptor interface. In our system, *B*^CS,CT^_for_ increases with fluorination of the donor (*i.e.*, reduced offset between the HOMO of the donor and the acceptor), which suggests that a reduced Δ*G*_CT,CS_ enhances the back transfer from the free charge carriers to the CT state. Unravelling the correlation between the energetics of the systems and the rate constant *B*^CS,CT^_for_ would help design more efficient OPV devices.

In this study we have found that when changing the HOMO energy of the donor, the LE dissociation rate constant as well as the CT dissociation and reformation rate constant are affected. These results challenge the common approach where changing the energy levels of the donor compared to the acceptor mainly impacts the transitions between the LE state and the CT state or the CT state recombination.^9,14^ If we assume that the dissociation and reformation rate constant are independent of the donor HOMO energy for the three devices considered (ESI,† Section 16), the lowest offset device would outperform the high offset device (14% PCE for the P4FBDB-T blend as compared to ∼10% PCE for the PBDB-T blend). If we assume that the CT dissociation rate constant and reformation rate constant are constant through the series (which also means fixing Δ*G*_CT,CS_), then the fill factor of the lowest offset blend would not be reduced and its *V*_oc_ would be higher than the experimental value and the result of the model used in the paper. The *J*_sc_ of the devices follow the same trend in this case as the experimental results, however the reduction in the *J*_sc_ in this case is mainly due to a reduced LE dissociation rate constant rather than a reduced CT dissociation rate constant as considered previously. These results confirm that changing the energy offset of the donor strongly affects the CT dissociation and reformation rate constants. It is therefore important to not only consider the energy offset between the LE and the CT state, but also the energy offset between the CT and the CS state.

In this work, we showed how through a unified model we can investigate the correlation between the reduced energy offset between the molecular orbitals of the donor and the acceptor and the rate of CT state dissociation and reformation. Although the model does not yet provide a predictive tool to relate chemical structure to device performance, we can use it as a tool to study and infer correlation between the model parameters and the material and device properties. This will help deepen our understanding of what controls the power conversion efficiency of OPV devices and establish design rules to improve it.

## Author contributions

M. A., N. P. G., F. E., X. Z., M. H., A. A. B. and J. N. conceived and planned the experiments, M. A., N. P. G., F. E., X. Z., H. C. and J. Y. carried out the experiments (NPG XZ and HC carried out the ultrafast measurements, M. A. and F. E. carried out the steady state and the transient optoelectronic measurements, J. Y. measured the transport properties of the films). M. A. developed and carried out the simulations. M. A. and N. P. G. wrote the manuscript with support from F. E., J. N. and A. A. B. Q. H., Z. F. and M. H. provided the molecules and helped with the interpretation of the results. All authors provided critical feedback and helped shape the research, analysis and manuscript.

## Conflicts of interest

There are no conflicts to declare.

## Supplementary Material

EE-015-D1EE02788C-s001
