# Supplementary material for: Reconciling models of interfacial state kinetics and device performance in organic solar cells: impact of the energy offsets on the power conversion efficiency
Source: Energy Environ Sci. 2022 Feb 7;15(3):1256–70. doi: 10.1039/d1ee02788c (PMC8924960; doi:10.1039/d1ee02788c)
Supplement: EE-015-D1EE02788C-s001 [file EE-015-D1EE02788C-s001.pdf]

Electronic Supplementary Material (ESI) for Energy & Environmental Science.
This journal is © The Royal Society of Chemistry 2022

Supporting Information:

Reconciling models of interfacial state kinetics and device performance in organic solar cells: Impact of the energy offsets on the power conversion efficiency

Mohammed Azzouzi [1,#] ,Nathaniel P. Gallop [2#], Flurin Eisner [1], Jun Yan [1], Xijia Zheng [2], Hyojung Cha[2,4]; Qiao He [2], Zhuping Fei [2,3], Martin Heeney [2], Artem A. Bakulin [2] and Jenny Nelson [1,*].*

[1] Department of Physics and Centre for Plastic Electronics, Imperial College London, London SW7 2AZ, United Kingdom.

[2] Department of Chemistry and Centre for Processable Electronics, Imperial College London, London W12 0BZ, United Kingdom

[3]Institute of Molecular Plus, Tianjin Key Laboratory of Molecular Optoelectronic Science, Tianjin University, Tianjin 300072, P.R.; China

[4] Department of Hydrogen & Renewable Energy, Kyungpook National University, Daegu, 41566, Republic of Korea

[#] Shared first author

[*] Corresponding Author:

jenny.nelson@imperial.ac.uk

Mohammed.azzouzi15@imperial.ac.uk

Table of Contents

Table of Contents 2
1. Methods 3
2. Model parameters: 6
3. Steady State Absorption and Energy levels of the materials 10
4. JV characteristic results 10
5. Lowest exciton properties: 11
6. Simulation of PL and EL in the simplified model. 13
7. CT state parameters 15
8. Effective density of states 15
9. Ultra fast kinetics 17
9.1. Transient absorption spectrum. 17
9.2. Global analysis details. 18
9.3. Modelling the GSB 19
9.4. Perfect reproduction of the GSB; case of PFBDB-T:C8-ITIC 20
10. Charge transport mobility estimated by space-charge-limited current method 23
11. Device model results : 29
11.1. Parameter exploration 29
11.2. TPV and TPQ 33
11.3. Fixed device parameters: 34
11.4. Key Model parameters changing along the series: 34
12. Device performances 35
13. Marcus rate for the dissociation rates of exciton and CT states. 35
14. Reformation rate constant and Langevin Encounter rate constant. 36
15. Improving the efficiency of low offset blends. 39
15.1. Case 1 : increasing both k_{dis}^{CT} and $B_{for}^{CS,CT}$. 39
15.2. Case 2 : increasing both kdisCT and ni 40
16. Case where the CT to CS state transition is independent of the energy offset. 40
17. References 44

1. METHODS

Materials and Synthesis

PBDB-T, (5-bromo-3-fluorothiophen-2-yl)tri(propan-2-yl)silane (1), 1,3-bis(5-bromo-4-fluorothiophen-2-yl)-5,7-bis(2-ethylhexyl)-4H,8H-benzo[1,2-c:4,5-c']bisthiophene-4,8-dione (9), PFBDB-T, P4FBDB-T and C8-ITIC were synthesized according to literature procedures [1,2].

Device Preparation and Characterization

All devices were fabricated in the inverse architecture on pre-patterned ITO covered glass substrates. After cleaning, ZnO was deposited from a zinc acetate anhydrous solution (110mg/ml in 2-methoxyethanol with 30μL ethanolamine per 1mL) by spin coating at 4000rpm, followed by annealing at 200 degrees. All blends were dissolved in chlorobenzene at a ratio of 1:1.25 D:A, and a concentration of 20mg/ml, and were stirred overnight at 50 degrees.. Blends were spin cast at spin speeds of 1500-2000rpm in a nitrogen atmosphere, and were soft-annealed at 80 degrees for 10 minutes after spin coating. The thickness of the active layers are around 80-100nm. To complete the devices, MoO_3 (10nm) and silver (100nm) were evaporated through shadow masks to yield devices with an area of 5mm^2.

External Quantum Efficiency Measurements

EQE measurements were carried out using a grating spectrometer (CS260-RG-4-MT-D) to create monochromatic light from a tungsten halogen light source. The light was chopped at 300Hz, and a Stanford Research System SR380 lock-in amplifier was used to detect the photocurrent. Long pass filters at 610, 780, 850 and 1000nm were used to filter out scattered light from the monochromator. The spectra were calibrated using a Silicon photodiode.

Electroluminescence and Photoluminescence Measurements

EL and PL spectra were recorded using a Shamrock 303 spectrograph combined with a iDUS InGaAs array detector which was cooled to -90°C. The obtained EL intensity spectra were calibrated with the spectrum from a calibrated Halogen lamp. A 473nm diode laser was used as the excitation source for PL spectra, which were measured using the same spectrograph and detector system as for EL measurements at the open-circuit voltage of the devices.

Drift-diffusion model.

A one-dimensional drift diffusion model was implemented to simulate the results using MATLAB's built-in partial differential equation solver for parabolic and elliptic equations (pdepe). The full details can be found in the reference . [3]. This model was used to simulate TPV, *J-V* characteristics and the delayed charge extraction measurements. For all the simulations we fixed the carrier densities at the boundaries to be the same as that of the transport layers in equilibrium.

TPQ and TPV was simulated using a symmetric model to allow the cell to be at open circuit conditions as described in Calado et al. [3] The laser length and light intensity were varied to keep the cell in the small perturbation regime, where the transient open circuit voltage did not exceed 30 mV. We used a uniform generation profile throughout the active layer of the device to simulate the bias light.

Optoelectronic characterization. (setup)

The TPV and TPQ experiment were carried in nitrogen atmosphere. Variable, continuous intensity illumination was provided using a ring of 1W white umiled LEDs incident on the

ITO side of the device. For the TPV experiment, once the device output reached a steady state, the device was perturbed using a single diffuse pulse from aPhoxX 638-150 - laser diode whose intensity was adjusted for every background light bias to assure that the system is on the small perturbation regime. The resulting voltage transient $\Delta V(t)$ ($\Delta V \ll V_{OC}$) was measured at the contacts using a DPO 5104B Tektronix digital phosphor oscilloscope. The 1 MΩ input impedance of the oscilloscope was used to hold the device at open-circuit throughout the measurement. Output transients were fitted to a single exponential function to obtain the time constant of the voltage decay. For the TPQ experiment, the cell was switch from open circuit to short circuit condition using a MOS-FET (ZVN4306A) that was controlled using a AFG3102C Tektronic pulse generator, the same signal from the pulse generator was used to control a MOS-FET (ZVN4306A) that switched off the LED ring. The current from the device was measured using the DPO 5104B Tektronix digital phosphor oscilloscope with a 46 Ω ohm resistance. The excess current was measured by subtracting the signal without a laser pulse, and the delay time between the laser pulse and the switch to short circuit was assured using the two output of the pulse generator.

J-V characterization:

Current-Voltage characteristics were measured using a Keithley 236 source-measure unit under AM1.5 solar irradiation (Oriel 300W solar simulator) at an intensity of 100mWcm-2. All electrical measurements were carried out in a nitrogen atmosphere.

General Experimental Details: Transient Absorption Spectroscopy

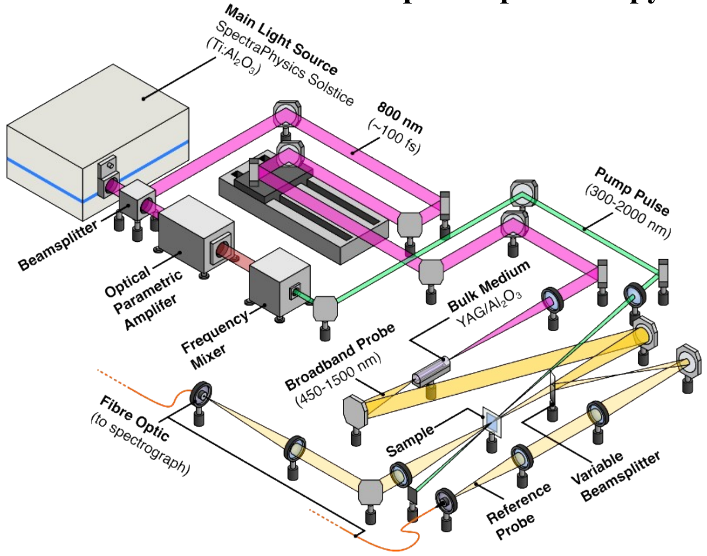

Figure S 1 Simplified schematic of the Transient Absorption spectrometer employed in this paper.

A diagram of the ultrafast transient absorption setup is provided in Fig. S4. The primary light source employed in this setup is a Ti:sapphire regenerative amplifier (Solstice, SpectraPhysics Inc.) operating at a repetition rate of 1 kHz, with a nominal temporal FWHM of c.a. 100 fs. A combination OPA/Frequency mixer (TOPAS, Light Conversion Inc.) is employed to create pump pulses in the 300—800 nm region. Ultra-broadband NIR (850—1450 nm) and visible (450—800 nm) probe pulses were created via bulk supercontinuum generation, respectively employing either a yttrium aluminium garnet (YAG) or sapphire crystal, respectively. The pump pulse was modulated at a frequency of 500 Hz using a mechanical chopper synchronised to the output of the primary light source. To introduce time resolution, the probe pulse was delayed by means of a mechanical delay stages. The pump and probe pulses were

independently focused onto the sample using a glass lens and spherical focusing mirror, respectively. The spot diameter of both the pump and probe pulses was approximately 500 μm at the surface of the sample. Prior to arrival of the probe at the sample, a 'reference' replica pulse was obtained using a variable reflective neutral density filter. Both probe pulse replicas were thereafter collimated and passed to a spectrograph, where their spectra were taken and stored. The resulting 2D maps were then processed using the SurfaceXplorer package (Ultrafast Systems Inc.) as well as home-built analysis software.

2. MODEL PARAMETERS:

Based on the principle of detailed balance we can calculate the radiative dark saturation current using [4,5]

$$J_{0,rad} = q\int A(\hbar\omega) * \phi_{bb}(\hbar\omega, T = 300\ K)d\hbar\omega \#(S1)$$

where $A(\hbar\omega)$ is the absorptance of the film, $\hbar\omega$ the energy of the photon ($\hbar$ is the reduced Planck constant, and ω the frequency of the photon) and ϕ_{bb} the blackbody spectrum at temperature T. Using equation 1, we can calculate the population density at equilibrium of both the CT ($[CT]_0$) and lowest exciton state ($[LE]_0$) considering that the recombination of the photoexcited species only occurs through the CT or LE states using:

$$J_{0,rad} = q * p_e * \left([CT]_0 * K^{CT}_{rec,rad} + [LE]_0 * K^{LE}_{rec,rad}\right) * d\#(S2)$$

where $K^{CT}_{rec,rad}$ and $K^{LE}_{rec,rad}$ are the radiative rate constants of the CT and LE respectively, d is the thickness of the device, q is the elementary electronic charge and p_e the emission probability. p_e can be calculated using the absorption coefficient and the absorptivity of the film and the refractive index (n) [4]. $[CT]_0$ and $[LE]_0$ are considered to follow Boltzmann statistics for the population of the states [6], therefore

$$\frac{[CT]_0}{[Ex]_0} = \exp\left(-\frac{\Delta G^0_{LE} - \Delta G^0_{CT}}{k_B T}\right) g_{CT,LE} \#(S3)$$

Where ΔG^0_{LE} and ΔG^0_{CT} are the free energies of the transition from the ground state to the LE and CT state, respectively. $g_{CT,LE}$ is the degeneracy ratio of CT to LE states [7]; k_B is the Boltzmann constant. Using the population of the states at equilibrium and the recombination rate constant from the states ($k^X_{rec} = K^X_{rec,r} + K^X_{rec,nr}$, where X is either CT or LE), we can calculate the dark saturation current

$$J_0 = q * \left([CT]_0 * k^{CT}_{rec} + [LE]_0 * k^{LE}_{rec}\right) * d.\#(S4)$$

Moreover, we can calculate the maximum short circuit current density under 1 sun condition using:

$$J_{sc,max} = \int A(\hbar\omega) * \phi_{AM\,1.5}(\hbar\omega)d\hbar\omega\ \#(S5)$$

Where $\phi_{AM\,1.5}$ is the Air Mass 1.5 photon flux in units [mA cm[2] eV[-1]]. Using equation [1-5] we can calculate the radiative voltage limit ($V_{oc,rad} = \frac{k_B T}{q} log(\frac{J_{sc,max}}{J_{0,rad}})$), the non-radiative voltage losses ($\Delta V_{oc,nr} = - \frac{k_B T}{q} log\left(\frac{J_{0,rad}}{J_0}\right)$) and an estimate open circuit voltage ($V_{oc} = V_{oc,rad} - \Delta V_{oc,nr}$) [8].

Table S 1 Example of model optical and state input parameter and the different model outputs.

	Input Parameter	Value/ Unit
**For each excited state**	Difference in the static dipole moment ($\lvert\Delta\vec{\mu}\rvert$)	10 Debye
	Oscillator strength (f_{osc})	1×10^{-3}
	High frequency reorganisation energy (λ_v)	0.13 eV
	Low frequency reorganisation energy (λ_l)	0.1 eV
	Vibrational mode harmonic oscillator energy ($\hbar\Omega$)	0.15 eV
	Free energy of the transition (ΔG)	1.65 eV
**Device as a whole**	refractive index (n)	1.5
	The ratio of CT states ($g_{CT,LE}$)	0.01
	CT state and S_1 coupling ($V_{S1,CT}$)	20 meV
	Effective density of LE states	$8\ 10^{19}$ cm^{-3}
	Thickness of the device (d)	10^{-5} cm
	**Model Output**	**Dependency**
**For each excited state**	Non radiative recombination rate constant $K^{S}_{rec,nr}$	$f(f_{osc}, \lambda_v, \lambda_l, \hbar\Omega, \Delta G, \lvert\Delta\vec{\mu}\rvert)$
	Radiative recombination rate constant $K^{S}_{rec,r} = \int k^{S}_{rec,r}(\hbar\omega)\, d\hbar\omega$	$f(f_{osc}, \lambda_v, \lambda_l, \hbar\Omega, \Delta G, \lvert\Delta\vec{\mu}\rvert)$
	Emission spectra ($k^{S}_{rec,r}(\hbar\omega)$)	$f(f_{osc}, \lambda_v, \lambda_l, \hbar\Omega, \Delta G, \lvert\Delta\vec{\mu}\rvert)$
	Population Density at equilibrium $[S]_0$	$J_{0,rad} = q * p_e * ([CT]_0 * K^{CT}_{rec,rad} + [LE]_0 * K^{LE}_{rec,r}$
**Device as a whole**	Absorptivity ($A(\hbar\omega)$)	$A(\hbar\omega) = 1 - \exp\left(-2d\alpha_{abs}(\hbar\omega)\right).$
	Absorption coefficient ($\alpha_{abs}(\hbar\omega)$)	$f(f_{osc}, \lambda_v, \lambda_l, \hbar\Omega, \Delta G, \lvert\Delta\vec{\mu}\rvert, V_{Ex}, d, g_{CT,LE}, n)$
	Radiative voltage Limit ($V_{oc,rad}$)	$V_{oc,rad} = \frac{k_B T}{q} log(\frac{J_{sc,max}}{J_{0,rad}})$
	Radiative dark saturation current ($J_{0,rad}$)	$J_{0,rad} = q\int A(\hbar\omega) * \phi_{bb}(\hbar\omega) d\hbar\omega$
	Maximum short circuit current under AM 1.5 ($J_{sc,max}$)	$J_{sc,max} = q\int A(\hbar\omega) * \phi_{AM\,1.5}(\hbar\omega) d\hbar\omega$
	Dark saturation current (J_0)	$J_0 = q * ([CT]_0 * k^{CT}_{rec} + [LE]_0 * k^{LE}_{rec}) * d$

	Non-radiative voltage losses ($\Delta V_{oc,nr}$)	$\Delta V_{oc,nr} = - k_B T \, log\left(\frac{J_{0,rad}}{J_0}\right)$

Table S 2 Kinetic model parameter, the rate constant for the different processes, with a distinction between the free input parameter and the dependent rate constants.

Kinetic model parameters		
Free Input Parameters		
Parameter	Symbol	Example Value / Unit
Exciton dissociation to CT state	$k_{dis}^{LE,CT}$	10^{11} s^{-1}
CT dissociation to free charge carrier	$k_{dis}^{CT,CS}$	10^{11} s^{-1}
Reformation rate constant from CS to CT	$B_{for}^{CS,CT}$	10^{-11} cm^{3}s^{-1}
Dependent parameters		
Parameter	Symbol	**Dependency**
Exciton reformation rate from CT state	$k_{trans}^{CT,LE}$	$\frac{k_{trans}^{CT,Ex}}{k_{dis}^{Ex,CT}} = \frac{[Ex]_0}{[CT]_0}$
Exciton recombination rate constant	k_{rec}^{Ex}	$k_{rec}^{Ex} = K_{rec,r}^{Ex} + K_{rec,nr}^{Ex}$
CT recombination rate constant	k_{rec}^{CT}	$k_{rec}^{CT} = K_{rec,r}^{CT} + K_{rec,nr}^{CT}$

Table S 3 Example device parameter for the drift diffusion parameter

Parameter name	Symbo l	Hole transfer layer	Intrinsic layer	Electron transfer layer
**Input device parameter**				
**Dielectric constant**	ε_s	3	3	3
**n-type acceptor density**	N_A	0	0	$4.10^{17}\ cm^{-3}$
**p-type donor density**	N_D	$4.10^{17}\ cm^{-3}$	0	0
**Thickness**		$100\ nm$	d	$100\ nm$
**Electron mobility**	μ_e	$10^{-3}\ cm^2V^{-1}s^{-}$	$10^{-4}\ cm^2V^{-1}s$	$10\ cm^2V^{-1}s^{-1}$
**Hole mobility**	μ_p	$10\ cm^2V^{-1}s^{-1}$	$10^{-4}\ cm^2V^{-1}s$	$10^{-3}\ cm^2V^{-1}s$
**Effective DOS in the conduction and valence bands**	N_{CB}, N_{VB}	$2.10^{19}\ cm^{-3}$	$2.10^{19}\ cm^{-3}$	$2.10^{19}\ cm^{-3}$
**Electron affinity**	Φ_{EA}	0	0	**0**
**Measurement parameters**				
**Uniform Generation rate under 1 sun Light intensity**	**G$_{av}$**	0	$\frac{J_{sc,max}}{q\ d}$	0
**Ionisation Potential**	Φ_{IP}	$\Phi_{IP} = - E_{CS} = - 2\ k_B T \log\left(N_{CB}/n_i\right)$		
**intrinsic charge carrier density**	n_i	$B_{for}^{CS,CT} * n_i^2 = [CT]_0 * k_{dis}^{CT}$		

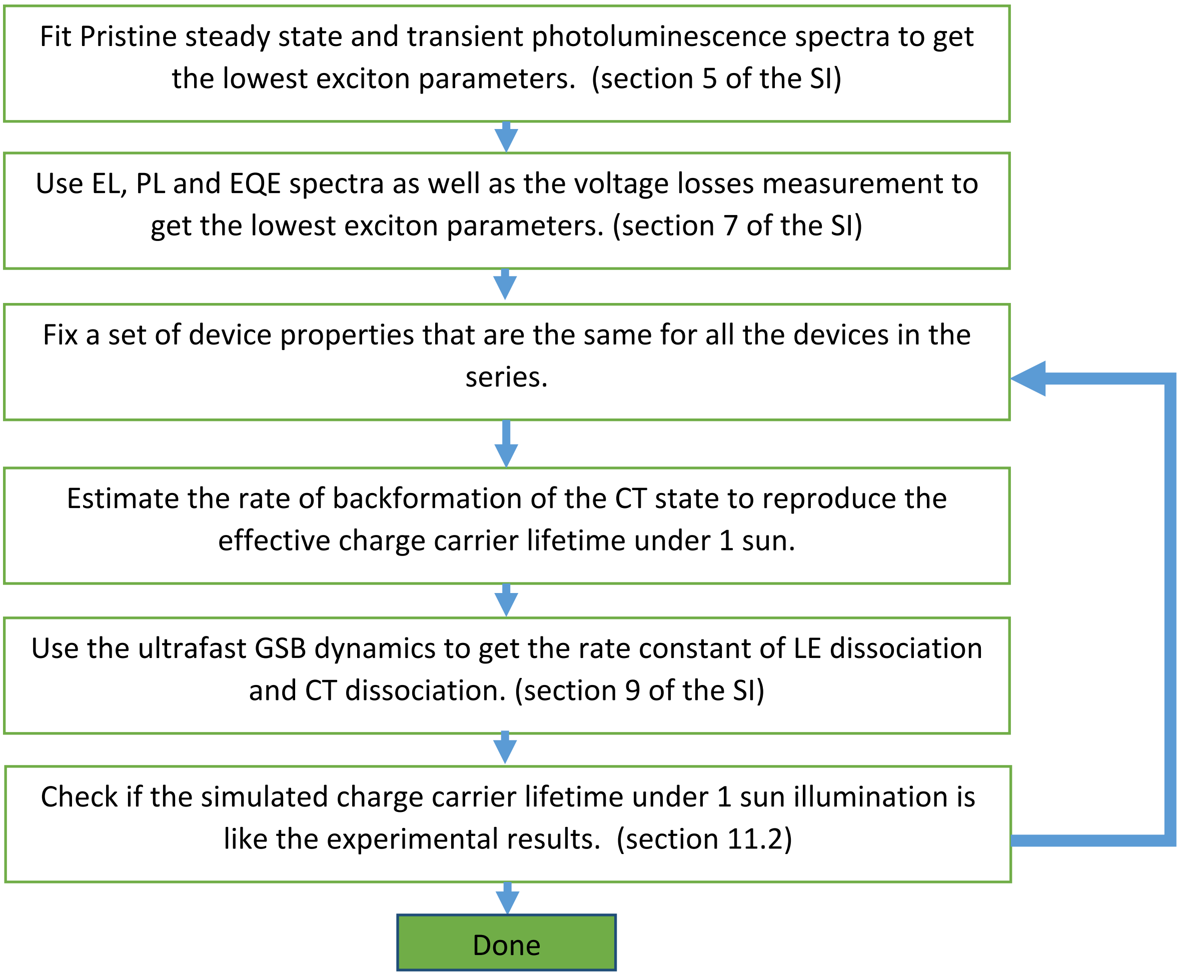

Diagram S1 Workflow used to estimate the different parameters of the model.

3. STEADY STATE ABSORPTION AND ENERGY LEVELS OF THE MATERIALS

Table S 4 Energy levels of materials obtained by cyclic voltammetry and air photoemission spectroscopy. All values are in eV from ref [2]

Material	E_{gap} (EQE)	HOMO (CV)	LUMO (CV)	HOMO (APS)
PBDB-T	1.83	-5.33	-3.29	-4.77
PFBDB-T	1.78	-5.47	-3.46	-4.85
P4FBDB-T	1.83	-5.63	-3.52	-4.95
C8-ITIC	1.53	-5.63	-3.91	

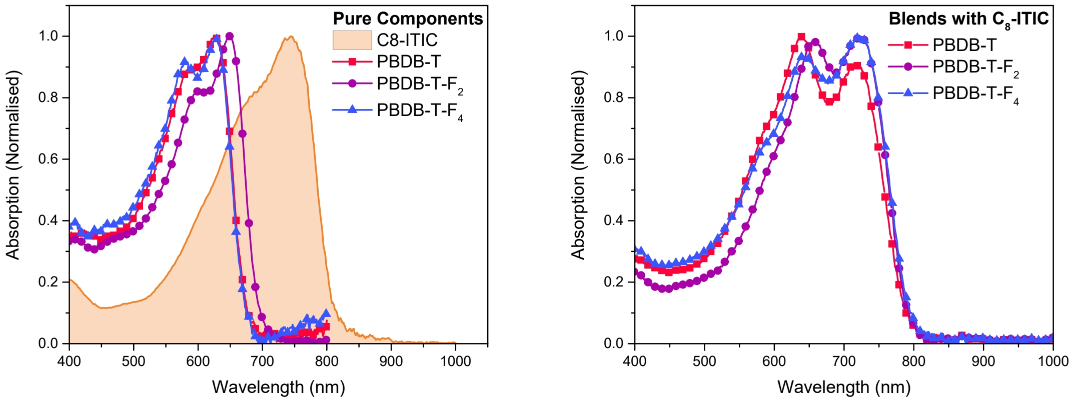

Figure S 2 Normalised absorption spectra of films of the pristine donor and acceptor species (A), and blends (B).

4. JV CHARACTERISTIC RESULTS

Table S 5 Device performances and transient optical characterisation results:

	Donor	PBDB-T	PFBDB-T	P4FBDB-T
Device performances	V_{oc} $[V]$	0.85	0.92	1.02
	J_{sc} $[mAcm^{-2}]$	17	17.5	12.5
	FF	0.64	0.65	0.59
	PCE (%)	8.12	10.26	7.42

5. LOWEST EXCITON PROPERTIES:

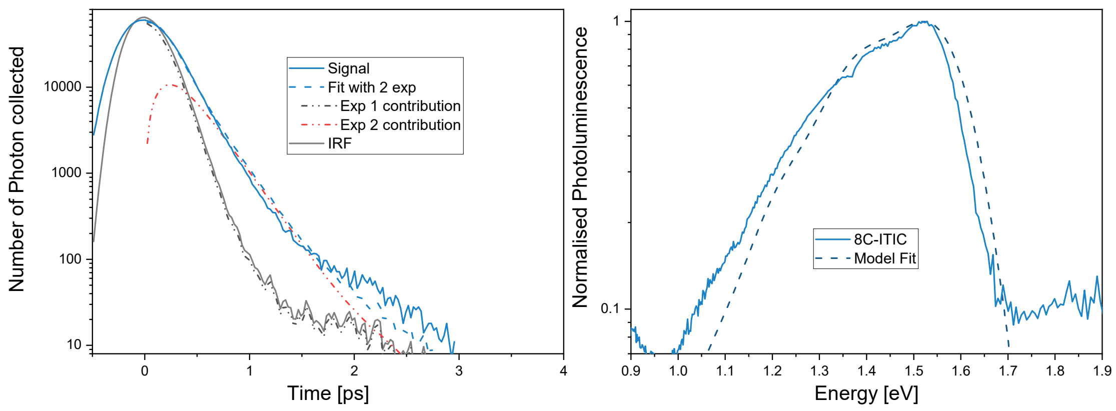

Figure S 3 A) Time correlated single photon counting (TCSPC) signal of the photoluminescence decay of pristine 8C-ITIC on a film measured at 950 nm and excited with a laser at 780 nm. The total model fit is a convolution of the IRF and a biexponential decay. B) PL of 8C-ITIC, excited with a laser at 410nm and a fit for the exciton PL using the parameters in table S6.

We measured the photoluminescence and transient photoluminescence of a film of pristine 8C-ITIC. The time correlated photon counting (TCSPC) of the luminescence of the film following an excitation with a laser pulse at 780 nm is shown in figure S2 a. The signal was fitted using a convolution of the IRF (measured at the same wavelength and under similar conditions) with a bi exponential decay function :

$$A_1 * \exp\left(-\frac{t}{\tau_1}\right) + A_2 * \exp\left(-\frac{t}{\tau_2}\right) .\# \ (S6)$$

The first exponential decay can be assigned to any process that occurs faster than the time resolution of our setup, (it mainly controls the first fast drop in the signal). The second decay can be assigned to the recombination of the exciton. The lifetime of the exciton is ($\tau_2 = 0.25\ ns$) which gives a recombination rate constant ($K_{rec} = \frac{1}{\tau_2} = 4\ 10^9\ s^{-1}$). Using the model for the recombination of the exciton introduced in the main text, we choose the parameters of the LE state to best reproduce the PL spectra of the device (Figure S2 b) and the lifetime of the exciton. The parameters as well as the model results are shown in table S6. The difference in the static dipole moment and the effective vibrational mode harmonic oscillator energy and the oscillator strength are fixed to arbitrary values that are common for similar systems. Free energy of the transition and the reorganisation energies are changed to best reproduce the experimental results.

Table S 6 Parameters to model the LE state in the blends (i.e. the lowest excited state in 8C-ITIC) and the model calculate recombination rate constant.

	Input Parameter	Value/ Unit
**Model Parameters**	Difference in the static dipole moment ($\lvert\Delta\vec{\mu}\rvert$)	10 Debye
	Oscillator strength (f_{osc})	5
	High frequency reorganisation energy (λ_v)	0.15 eV
	Low frequency reorganisation energy (λ_l)	0.1 eV
	Vibrational mode harmonic oscillator energy ($\hbar\Omega$)	0.15 eV
	Free energy of the transition (ΔG)	1.63 eV
**Model results**	Non radiative recombination rate constant $K_{rec,nr}^{LE}$	4.9 10^9 s^{-1}
	Radiative recombination rate constant $K_{rec,r}^{LE} = \int k_{rec,r}^{LE}(\hbar\omega)\, d\hbar\omega$	4.9 10^8 s^{-1}

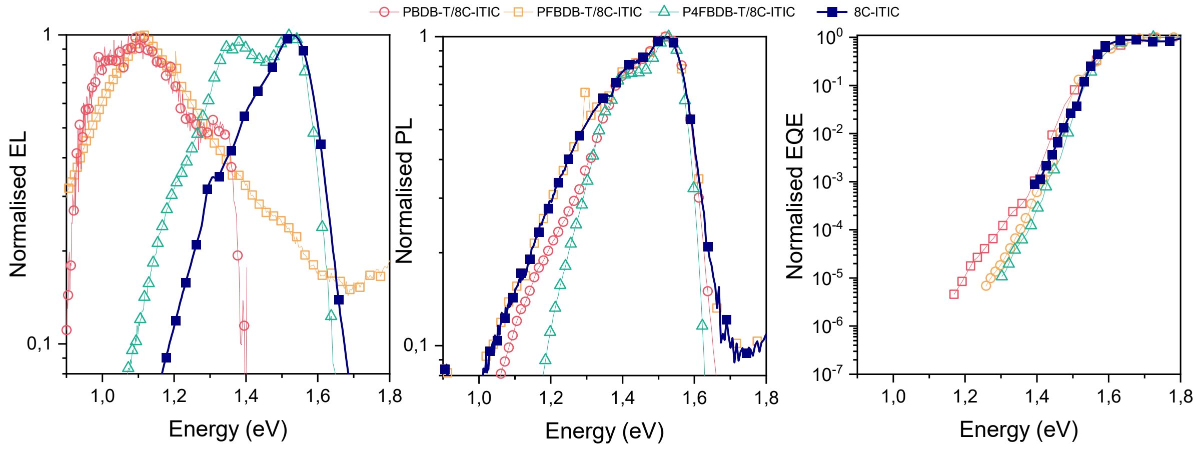

Figure S 4 Steady state optical properties of the blends and the pristine 8C-ITIC. a) Normalised electroluminescence spectra of the three blend devices at an injection current of 100 mA cm^{-2}. b) Normalised Photoluminescence spectra of the devices. c) Normalised EQE of the three blends.

6. SIMULATION OF PL AND EL IN THE SIMPLIFIED MODEL.

To simulate the luminescence of the devices under different conditions, we need to estimate the population of the LE and CT states (considering they are the only excited states that can recombine radiatively); and calculate

$$\phi_{em}(\hbar\omega) = [CT]_{sum} * k_{rec,r}^{CT}(\hbar\omega) + [LE]_{sum} * k_{rec,r}^{LE}(\hbar\omega) \#(S7)$$

The emission flux per energy of the device (units $cm^{-2} s^{-1} eV^{-1}$). $[CT]_{sum}$ is the sum of the population of CT state in the active layer ($[CT]_{sum} = \int [CT]dx$). To calculate ϕ_{em} we need to simulate the device under a specific applied electrical bias and light bias. For EL, the device is held under dark and we apply a positive voltage bias. For the PL we first keep the device under open circuit and under a specific light intensity. We can also simulate the EL and PL in a simpler case, where we neglect the effect of transport and only simulate the kinetic model in a 0D approximation. The kinetic model in question would be :

$$\frac{d[LE]}{dt} = G - k_{dis}^{LE,CT}[LE] + k_{trans}^{CT,LE}[CT] - k_{rec}^{LE}([LE] - [LE]_0)\#(S8)$$

$$\frac{d[CT]}{dt} = k_{dis}^{LE,CT}[LE] - k_{dis}^{CT}[CT] + B_{for}^{CS,CT}([n][p]) - k_{rec}^{CT}([CT] - [CT]_0) - k_{trans}^{CT,LE}[CT]\#(S9)$$

$$\frac{d[n]}{dt} = G_{inj} + k_{dis}^{CT}[CT] - B_{for}^{CS,CT}([n][p])$$

$$\frac{d[p]}{dt} = G_{inj} + k_{dis}^{CT}[CT] - B_{for}^{CS,CT}([n][p])\#(S10)$$

Where G_{inj} the generation of free charge replace $\frac{1}{q}\frac{\partial J_n}{\partial x}$. This approximation is only valid when we consider the device under low injection conditions, and that the impact of the field and the charge distribution is negligible. In this case, the EL would be simulated by considering (G_{inj}=10^{22} and G=0), and the PL in the case where (G_{inj}=0 and G=10^{22}).

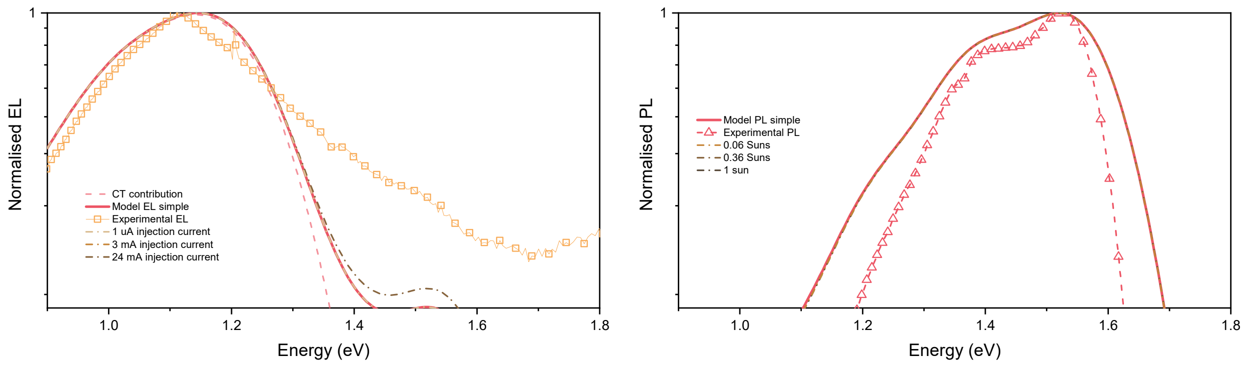

Figure S 5 a) Modelled EL at different injection current and considering the simplified case with no transport and a fixed injection of the free charge carriers. B) Modelled PL using the full drift diffusion model at different light generation intensities compared to both the experimental results for D0:A2 and the results of the simple model.

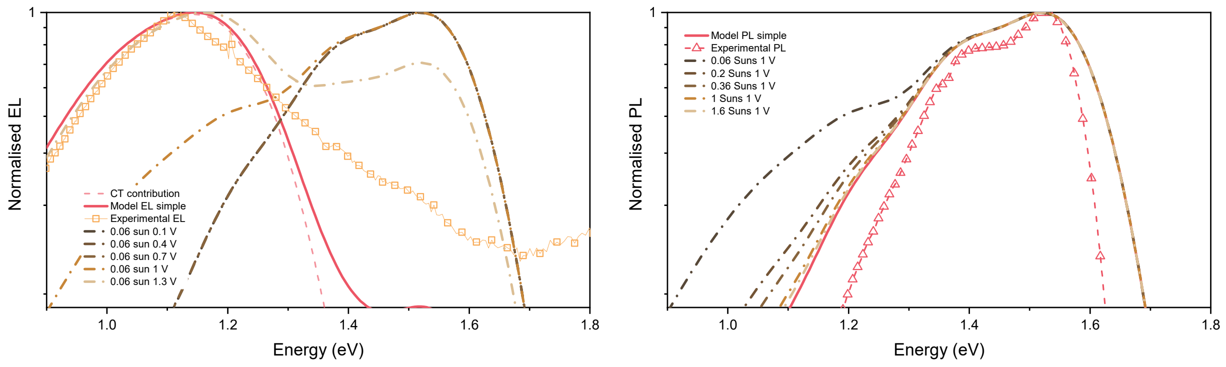

Figure S 6 the interplay between the light generation and the electric injection and their impact on the modelled EL and PL.

7. CT STATE PARAMETERS

Disclaimer: In the introduction of the model, we have introduced a set of free parameters (more than 15 just for the first part of the model (figure 1 c)), and these free parameters can impact the experimental results in similar ways [9]. Considering that these parameters are hard to accurately calculate for the systems studied [10], we rely on the experimental results as well as a set of constraint for the choices of the free parameters. The parameters chosen in this study are therefore not unique and are only valid considering our set of approximations and assumptions.

Table S 7 CT state and optical parameters considered for the reproduction of the Experimental data of the series.

Input Parameter		Value/ Unit
**For the CT state**	Difference in the static dipole moment ($\lvert\Delta\vec{\mu}\rvert$)	10 Debye
	Oscillator strength (f_{osc})	5×10^{-4}
	High frequency reorganisation energy (λ_v)	0.15 eV
	Low frequency reorganisation energy (λ_l)	0.15 eV
	Vibrational mode harmonic oscillator energy ($\hbar\Omega$)	0.15 eV
	Free energy of the transition (ΔG)	[1.31,1.34,1.42] eV
**Device as a whole**	refractive index (n)	1.5
	The ratio of CT states ($g_{CT,LE}$)	0.5
	CT state and LE coupling ($V_{LE,CT}$)	1 meV
	Effective density of LE states (N_{LE})	8 10^{19} cm^{-3}
	Thickness of the device (d)	10^{-5} cm

8. EFFECTIVE DENSITY OF STATES

In this section we review the effective density of the CT and LE states resultant from the assumptions of the model. The value of N_{LE} initially considered in the model was to ensure that the absorption coefficient of the device is high enough (absorption coefficient around 2 10^5 cm^{-1} close to the band gap of the material).. In the model we calculate the population density of the CT and LE states based on the reciprocity principle and the radiative recombination rate constate for the two states (equation S2). We also consider that both the CT and LE state follow a Boltzmann distribution and are in thermal equilibrium. The population density of CT and LE states under a common chemical potential (ϕ) can be written as:

$$[CT] = N_{CT}\exp\left(-\frac{\Delta G_{0,CT} - \phi}{k_B T}\right) \, and \, [LE] = N_{LE}\exp\left(-\frac{\Delta G_{0,LE} - \phi}{k_B T}\right) \#(S\ 11)$$

Where N_{CT} and N_{LE} are the effective density of states of the CT and LE state, respectively. Considering that at equilibrium $\phi = 0$; we can calculate N_{CT} and N_{LE} using the density of LE and CT state at equilibrium ($[LE]_o$ and $[CT]_o$, respectively). This equation offers a different way of calculating N_{LE} that is independent of the one considered in the model. In a first instance we consider that the energy of the states is equal to the free energy of the transition (ΔG_0). The back calculated N_{LE} in this case agrees with the one considered in the model for the three blends in the paper.

9. ULTRA FAST KINETICS

9.1. Transient absorption spectrum.

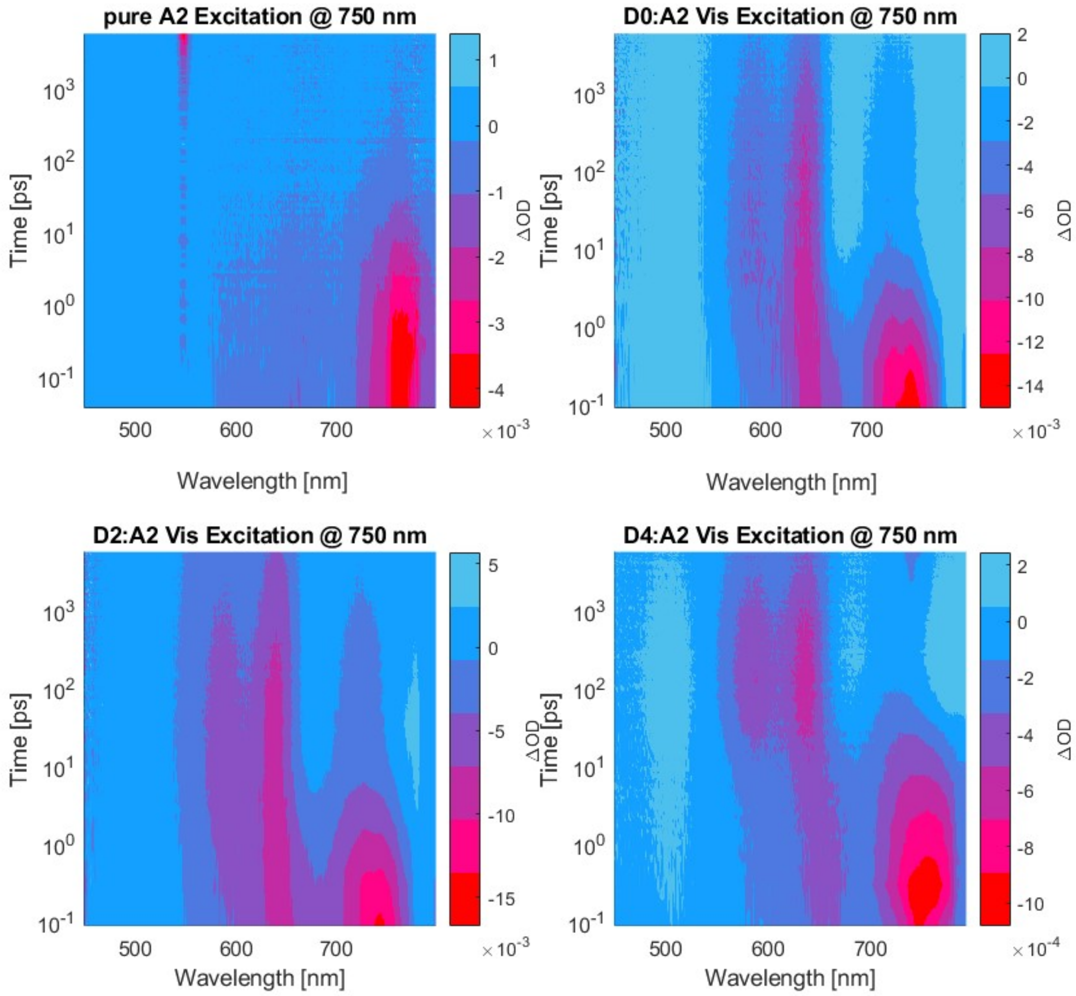

Figure S 7 Two-dimensional transient absorption maps of (A) C_8-ITIC, (B) PBDB-T/C_8-ITIC, (C) PFBDB-T/C_8-ITIC, and (D) P4FBDB-T/C_8-ITIC. Laser Excitation wavelengths of 750 nm and power of 20 nJ.

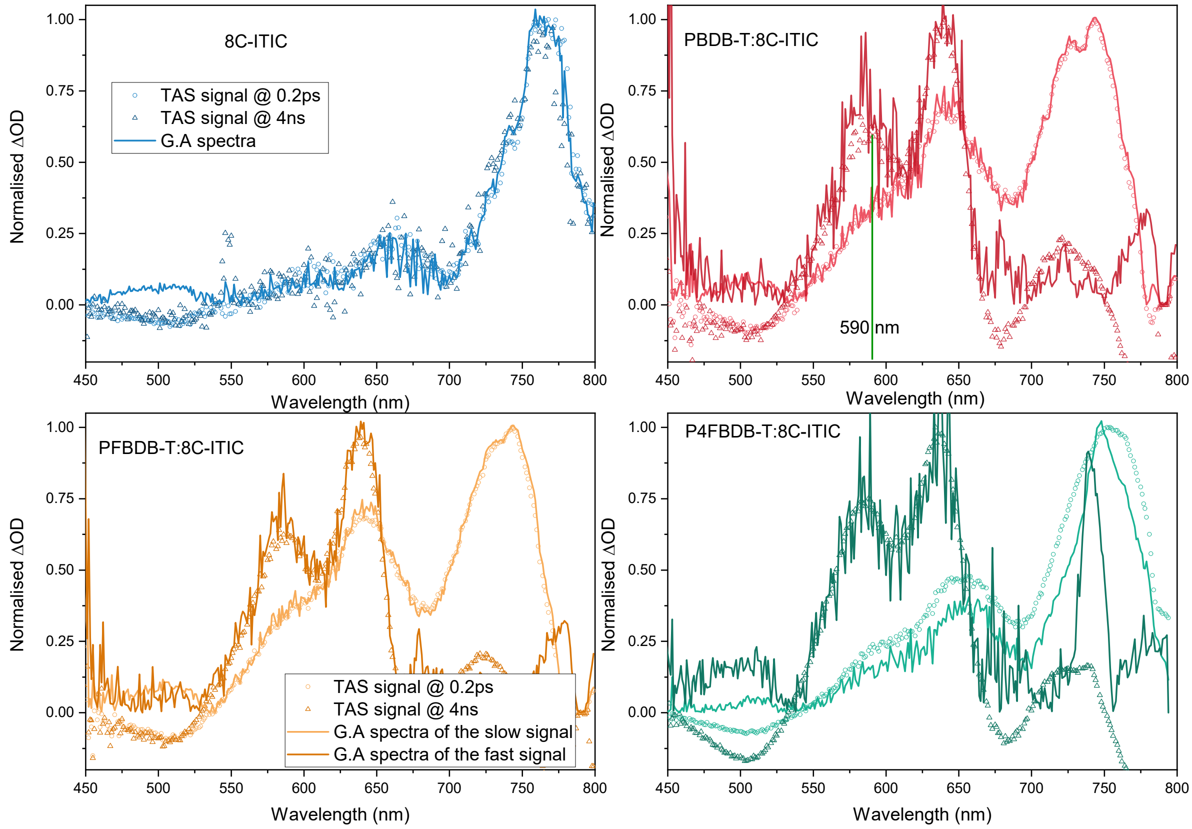

Figure S 8 •A plot with the spectra from the GA and the early and long time for the three blend; (to show why we chose the wavelength to plot and associate with GSB in the main text).

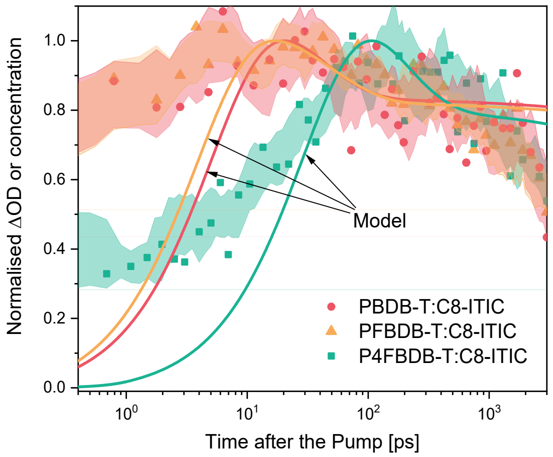

Figure S 9 • Dynamics considered from the model and the differences between the three devices.

9.2. Global analysis details.

To analyse the transient absorption spectrum, we used the global analysis toolbox developed by Van Wilderen et al. [11] considering: one species for the pristine acceptor with a mono-exponential decay; Two species in sequence for the blends, where they both decay monoexponentially, and the decay of the fast specie populates the slow one. The results of the

Global analyse spectra are shown in figure S8 to accurately reproduce the fast and slow transient absorption signal.

9.3. Modelling the GSB

The modelling of the GSB is done considering that the GSB signal is proportional to the population of free charges and CT states in the active layer ([CT], [n] and [p]). The modelling is done by considering the device in the dark and under open circuit condition. The open circuit voltage in the drift diffusion model is insured using the approach developed by Calado et al. using a symmetric device [3,12]. After solving for the condition where the device is in the dark and under open circuit condition, we solve the system considering that after 1 ps, a 0.2 ps laser pulse, generate LE state uniformly across the active layer with intensity equivalent to 1000 suns. After the laser pulse, the device is back in the dark and under open circuit conditions. From the model we can extract the population density of the electrons, holes and CT states in the active layer. We calculate the population densities by summing over the length of the active layer and consider that to be equivalent to the signal of the GSB.

The GSB can also be simplified considering a reduced impact of the transport as discussed in the EL and PL case section 5 of this document. We can just solve the kinetic model in equations (S3-5) considering a small generation laser pulse. Figure S10 shows a comparison between the two model and how they both agree well in the early times and only start differing after 1 ns. We use the simple model for the initial fitting procedure.

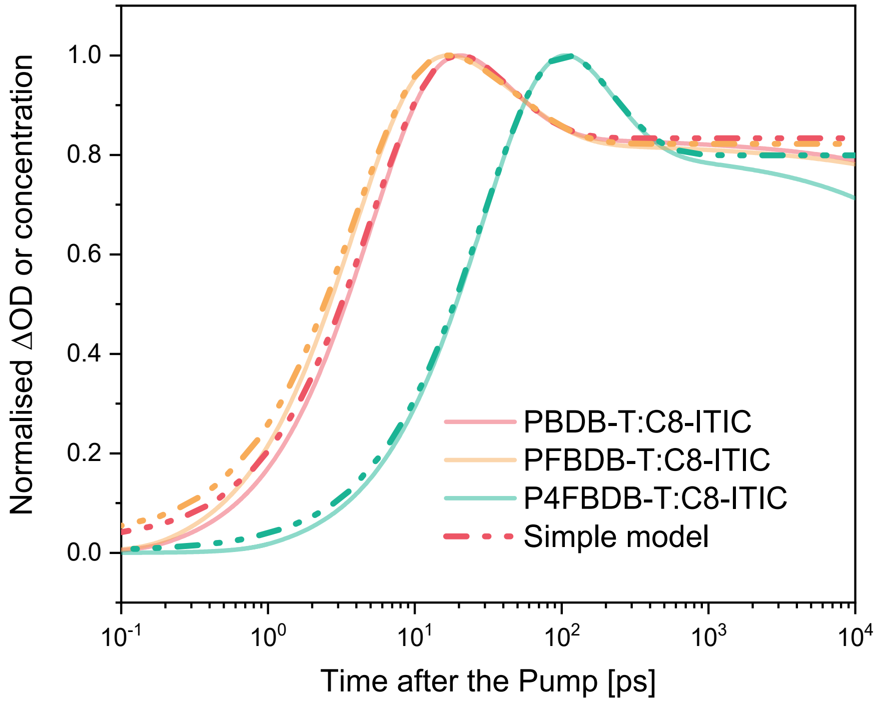

Figure S 10 Comparison of the model results considering the full drift diffusion simulation and the simple model without transport.

Table S 8 Kinetic model parameters Used to reproduce the GSB data, the only fitting parameters proper to the GSB dynamics are the rate of dissociation of the LE and CT state.

	Donor	PBDB-T	PFBDB-T	P4FBDB-T
**Model parameter**	$k_{rec}^{CT}[ns^{-1}]$	7	5	2
	$B_{for}^{CS,CT}[cm^3s^{-1}]$	$2\ 10^{-11}$	$1.3\ 10^{-10}$	$7.4\ 10^{-10}$
	$k_{trans}^{CT,LE}[ns^{-1}]$	$1.5\ 10^{-3}$	$6.6\ 10^{-3}$	$1.5\ 10^{-2}$
	$k_{rec}^{LE}[ns^{-1}]$	5	5	5
**Fitting Parameters for the GSB signal**	$k_{dis}^{LE,CT}[ns^{-1}]$	170	240	25
	$k_{dis}^{CT,CS}[ns^{-1}]$	20	16	4.1

9.4. Perfect reproduction of the GSB; case of PFBDB-T:C8-ITIC

Considering the difference observed between the model considered in the paper and the GSB data inferred from the global analysis (figure 5 in the main text). We discuss in this section the impact of a perfect reproduction of the GSB data on the device performances.

First, we noticed that the dynamics of the GSB after the first rise in the case where the reformation of the exciton is considerably slow compared to the other processes, is mainly related to the dissociation ,recombination and reformation of the CT state. In order to best reproduce the GSB data, we can adopt 2 different approaches, 1) reduce the CT recombination rate constant or 2) increase the CT reformation rate constant.

For the first case we need to then modify the properties of the CT state (i.e. its energy and reorganisation energies); the CT state properties are modified in a way to ensure that the recombination rate constant is in the order of 20 ns^{-1} which is an order of magnitude faster than the one we used in the paper. The impact of changing the CT energy and its reorganisation energy on the EL, PL and EQE is presented in figure S11. From Figure S11, the luminescence and absorption properties of the device can be reproduced significantly well with the new parameters. The fit to the GSB data is presented in figure S12, and as expected the new model better reproduces the hole GSB signal. However, when we use the model parameters and simulate the JVs, we find that the J_{sc} is significantly lower, and this is due to the slow dissociation rate constant that is needed to reproduce the GSB data. The open circuit and FF for this case is higher than the one used in the model, this is related to the slower recombination rate constant of the CT state and the slower reformation rate constant.

For the second case, we consider that the decay in the GSB is related to a fast reformation of the CT state from the free charge carriers. For this case, the properties of the CT state are similar to the one considered in the main text, but the reformation rate constant is considered

to be 6 orders of magnitude higher to better reproduce the GSB decay (red line in figure S10). The impact of this change is rather detrimental to the device performances, as the J_{sc} and FF drops significantly due to the high recombination rate of the free charge carries.

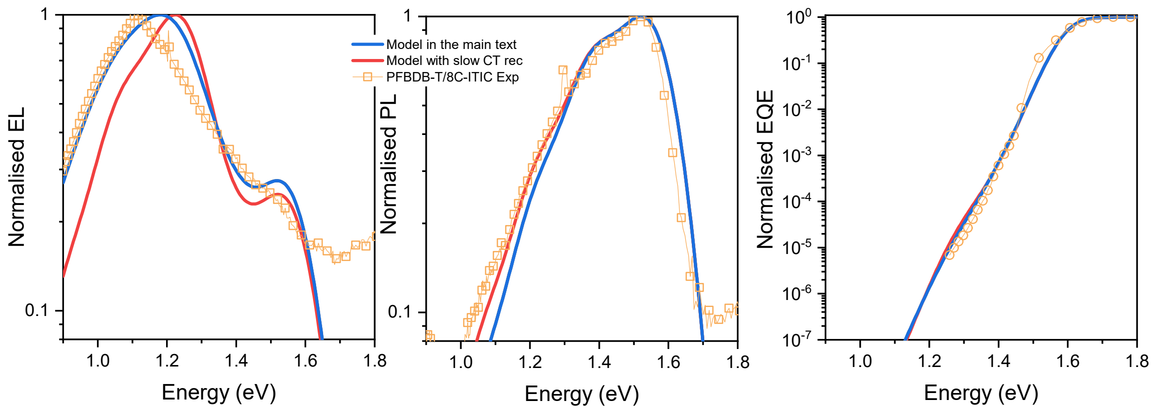

Figure S 11 Experimental EL, PL and EQE of the PFBDB-T:C8-ITIC blend, and the results of the different model considered for the reproduction of the experimental results. The case discussed in the main text considers the parameter in table S7 and the case of slow CT recombination in table S11.

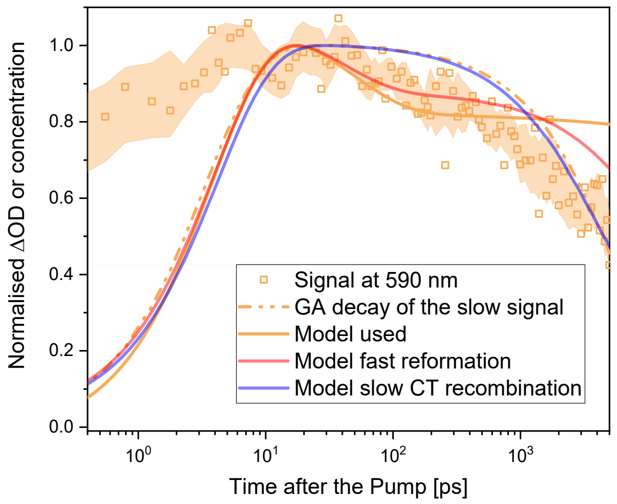

Figure S 12 GSB model and experimental results for the PFBDB-T:C8-ITIC blend with three different model as described in the text.

Table S 9 Processes rate constants calculated and estimated for the three different models.

	$K_{rec,nr}^{CT}$ (s^{-1})	$K_{rec,r}^{CT}$ (s^{-1})	$B_{for}^{CS,CT}$ ($cm^3\,s^{-1}$)	$k_{dis}^{CT,CS}$ (s^{-1})	$k_{dis}^{LE,CT}$ (s^{-1})	Offset (eV)	ΔG_0^{CT} (E_{CT}) (eV)
**Model in the paper**	**4.8 10^9**	**3.8 10^4**	**1.2 10^{-10}**	**1.5 10^{10}**	**2.0 10^{11}**	**0.29**	**1.34**
**Slow CT recombination**	**2.0 10^8**	**1.3 10^4**	**1.5 10^{-11}**	**7 10^7**	**2.3 10^{11}**	**0.31**	**1.32**
**Fast CT reformation**	**4.8 10^9**	**3.8 10^4**	**2 10^{-5}**	**1.5 10^{10}**	**2.0 10^{11}**	**0.29**	**1.34**

Table S 10 Device performance modelled for the three different models of PFBDB-T:C8-ITIC device.

	V_{oc_rad} (V)	J_{sc} (mA cm^{-2})	V_{oc} (V)	FF	E_{CS}
**Model in the paper**	**1.26**	**17.03**	**0.92**	**0.66**	**1.28**
**Slow CT recombination**	**1.26**	**6.17**	**0.98**	**0.76**	**1.34**
**Fast CT reformation**	**1.26**	**0.74**	**0.92**	**0.33**	**1.58**

Table S 11 CT state properties modified for the slow CT recombination case.

	**Input Parameter**	**Value/ Unit**
**For the CT state**	Oscillator strength (f_{osc})	3×10^{-4}
	High frequency reorganisation energy (λ_v)	0.1 eV
	Low frequency reorganisation energy (λ_l)	0.12 eV
	Free energy of the transition (ΔG)	1.32 eV

10. CHARGE TRANSPORT MOBILITY ESTIMATED BY SPACE-CHARGE-LIMITED CURRENT METHOD

1. Device fabrication and J-V measurements

The device structure for electron only device is ITO/ZnO(40nm)/Active layer/BCP(10nm)/Ag(80nm), and for hole only device is ITO/PEDOT:PSS(40nm)/Active layer/MoO3(10nm)/Ag(80nm). Poly(3,4-ethylenedioxythiophene): poly(styrenesulfonate) (PEDOT:PSS) (Clevios PPVP AI 4083, H.C Starck Inc.) was spin-cast on the precleaned ITO anode substrate and then dried in air at 150°C for 20 min, giving a thickness of 40 nm. Bathocuproine (BCP) was evaporated using organic source in vacuum chamber with a thickness of 10 nm. The processing conditions for active layer, ZnO, MoO3, and Ag follow the same procedure of solar cell fabrication in the method section. All devices were measured with a voltage range of -5 V to +5 V using Keithley 236 under dark.

2. Estimated band and effective mobilities using trap-limited transport model

Space charge limited current method has often been used to extract the mobilities of organic solar cells. This method allows us to obtain mobility values via simply fitting the slope 2 region of the experimental data following Mott-Gurney law, i.e. $\frac{\log(J)}{\log(V)} = 2$. However, in disorder semiconductor-based devices, Mott-Gurney law is no longer applicable as trap limited transport plays a significant role.

Here we adapt the method of numerical fitting using drift-diffusion model coupled with exponential tail states, which explicitly includes the effect of trap states and has been proved to be useful for organic solar cells[1,13,14]. Shown in Figure S13 and S14 are measured and simulated J-V and $\frac{\log(J)}{\log(V)}$ slope characteristics for electron- and hole-only devices, respectively. As we can see that a slope of 2 is rarely seen in the devices measured, indicating that Mott-Gurney law cannot be used in the analysis here.

In the fitting process, we aim to fit the current density under both negative and positive bias for each device, which are indicated by the current density difference in the J-V curves. We consider five fitting parameters as shown in Table S12, within which the trap-free mobility ($\mu_{n,p}$), characteristic energy for exponential tail ($E^{u}_{n,p}$), and effective trap density ($U^{exp}_{n,p}$) are of the most importance. The extracted values of $\mu_{n,p}$, $E^{u}_{n,p}$, and $U^{exp}_{n,p}$ are shown in Table S13. The results show that D2F:C8-ITIC blend presents the highest electron and hole (trap free) mobility compared to D0F:C8-ITIC and D4F:C8-ITIC blends. In general, trap free mobility is

less useful as in the operation condition the effective mobility is the one controlling charge transport. And effective mobility is determined by the charge carrier density inside the devices.

Since we have measured the free charge carrier density at 1 sun condition using charge extraction as presented in Figure 6 in the main text, we can estimate the effective mobility[15,16] (u_{eff}) at 1 sun condition using

$$u_{eff} = u_0 * \frac{n_f}{n_f + n_t}, (S12)$$

Where u_0 is the trap free mobility, and n_t is the trapped charge density in the exponential tail, given by[16]

$$n_t = U_t E_u \left(\frac{n_f}{N_C}\right)^{k_b T/E_u}, (S13)$$

The estimated values of effective electron and hole mobilities are shown in Table S13, showing that D2F:C8-ITIC blend has higher effective mobilities than the other two blends, especially in the case of hole transport, and the transport mobilities of D0F:C8-ITIC and D4F:C8-ITIC blends are like each other. We note here that the FF and Jsc of D0F:C8-ITIC and D4F:C8-ITIC blends are very similar (see Table S13), such differences on the effective hole transport mobilities (close to one order of magnitude difference) are inconsistent with the small device performance differences. Also, similar charge transport mobilities in D0F:C8-ITIC and D4F:C8-ITIC blends again don't agree with the difference on the FF and Jsc results in OPV devices. This indicates that early time scale process plays a more important role than charge transport processes. In addition, we have shown in the main text that the energetics between CT and CS states seem to be more important than mobilities in determining the reformation rate of CT excitons from free charges. Therefore, in the drift-diffusion modelling we consider that electron and hole mobilities are identical in each blend to rule out the effect of mobilities paying more attention on the early time scale processes, such as charge generation, exciton dissociation, and CT state exciton dissociation, CT state reformation from free charge carriers.

Table S 12. Input parameters for drift diffusion modelling using gpvdm[17]

Parameters	Symbol	Values	Units
Temperature	T	300	K
Relative dielectric constant	ε_r	3	unitless
Effective density of states of free charges	N_C/N_V	1×10^{26}	m^{-3}
Generation rate	G	0 (dark)	m^{-3} s^{-1}
Free to free charge recombination coefficient	B_{dir}	10^{-15}	m^3 s^{-1}
LUMO electron capture cross section	σ_e^e	1e-15	m^2
LUMO hole capture cross section	σ_h^e	1e-20	m^2
HOMO electron capture cross section	σ_e^h	1e-20	m^2
HOMO hole capture cross section	σ_h^h	1e-15	m^2
Effective band gap	E_g	Depending on the material	eV
Active layer thickness	L	Depending on the film	nm
Cathode injection barrier	φ_{cat}	Fitting parameters	eV
Anode injection barrier	φ_{an}	Fitting parameters	eV
Trap-Free Electron or hole Mobility	$\mu_{n,p}$	Fitting parameters	cm^2 $V^{-1}s^{-1}$
Effective electron or hole trap density	$U_{n,p}^{exp}$	Fitting parameters	m^{-3} eV^{-1}
Characteristic energy for electron or hole exponential tail	$E_{n,p}^{u}$	Fitting parameters	eV

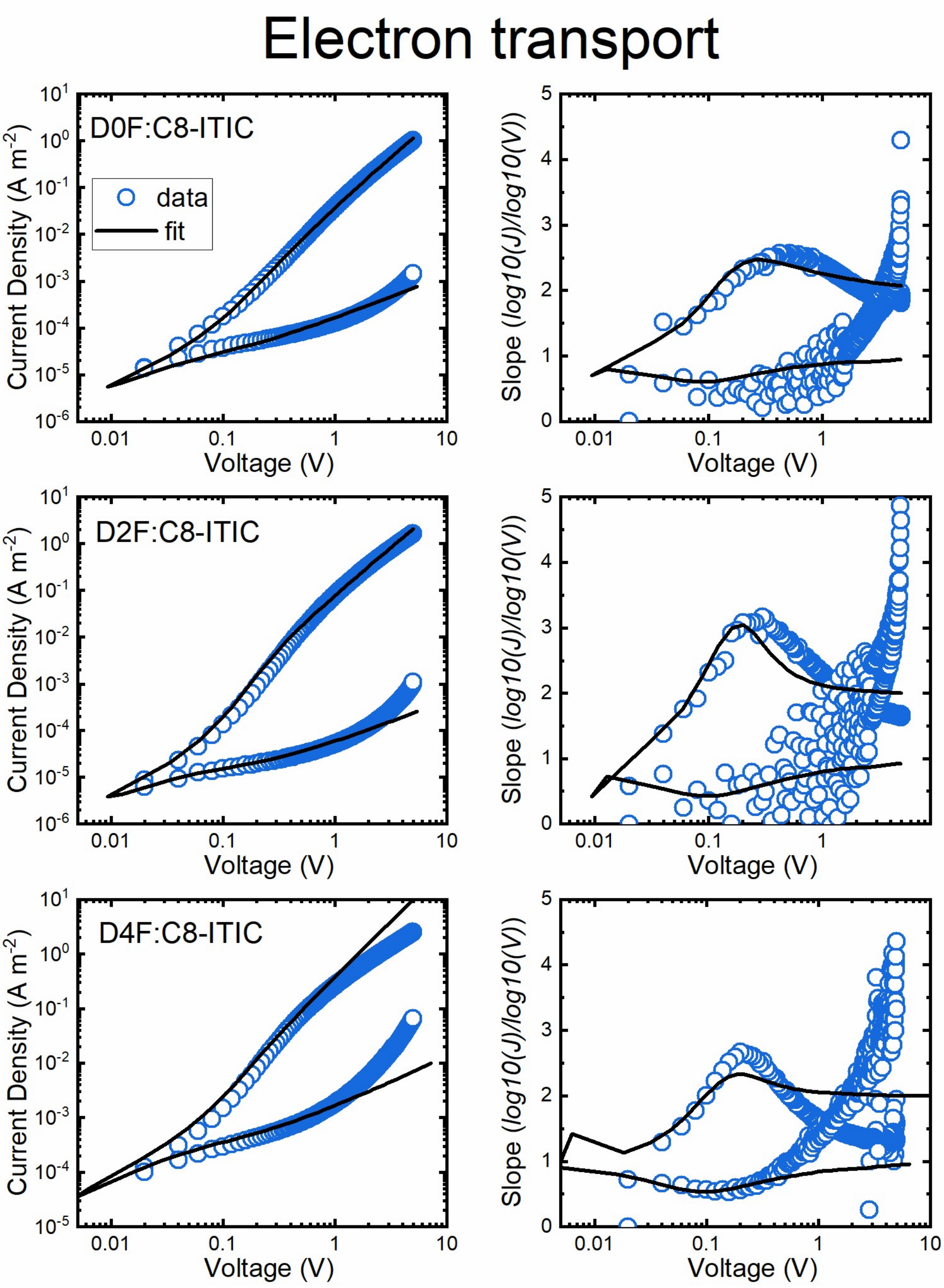

Figure S 13 Measured and simulated J-V characteristics of electron only device.

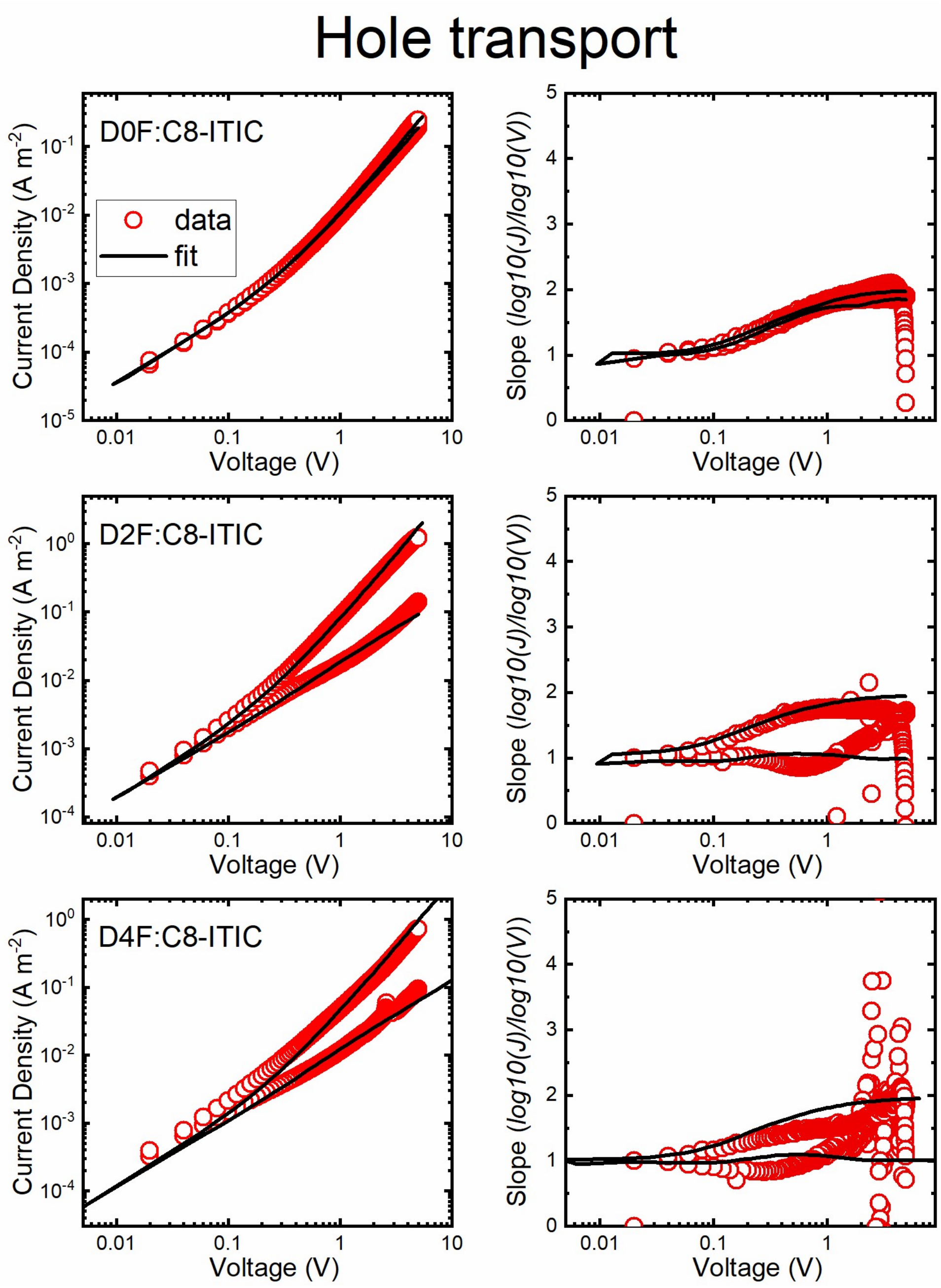

Figure S 14 Measured and simulated J-V characteristics of hole only device.

Table S 13 Extracted parameters using drift-diffusion modelling

Parameters	Trap-Free Electron Mobility	Effective electron trap density	Characteristic energy for electron exponential tail	Total trap density	Effective electron mobility @1 sun
**Symbol**	μ_n	U_n^{exp}	E_n^u	N_t	$\mu_{n,eff}$
**Units**	**$cm^2\ V^{-1}s^{-1}$**	**$m^{-3}\ eV^{-1}$**	**eV**	**m^{-3}**	**$cm^2\ V^{-1}s^{-1}$**
D0F:C8-ITIC-e	4.30E-04	3.50E+23	0.11	3.85E+22	3.73E-04
D2F:C8-ITIC-e	7.00E-04	2.70E+23	0.01	3.51E+21	7.00E-04
D4F:C8-ITIC-e	3.00E-04	2.00E+23	0.12	2.40E+22	2.42E-04

Parameters	Trap-Free Hole Mobility	Effective hole trap density	Characteristic energy for hole exponential tail	Total trap density	Effective hole mobility @1 sun
**Symbol**	μ_p	U_p^{exp}	E_p^u	N_t	$\mu_{p,eff}$
**Units**	**$cm^2\ V^{-1}s^{-1}$**	**$m^{-3}\ eV^{-1}$**	**eV**	**m^{-3}**	**$cm^2\ V^{-1}s^{-1}$**
D0F:C8-ITIC-h	8.10E-05	7.90E+23	0.09	7.11E+22	6.82E-05
D2F:C8-ITIC-h	5.60E-04	7.90E+23	0.05	3.95E+22	5.47E-04
D4F:C8-ITIC-h	2.50E-05	2.70E+23	0.06	1.62E+22	2.44E-05

11. DEVICE MODEL RESULTS :

11.1. Parameter exploration

Using the kinetic model presented previously, we have explored the effect of the different rate of transition between the states and other parameters on the Jsc, FF, Voc, lifetime of the charge carriers and the TPV lifetime. The parameters explored as well as the properties of the device considered are summarised in table S14. Here we used the model to simulate the current voltage characteristic of the device to extract the Jsc, FF and Voc , and simulated the TPQ and TPV measurement to extract the lifetime τ_Q and τ_{TPV}.

The short circuit current depends strongly on the ratio $\frac{k_{dis}^{CT}}{k_{rec}^{CT}}$ as the generation of free charge carriers is related to how fast the formed CT states can dissociate before recombining. Figure S15 shows the dependence of the simulated J_{sc} on the ratio $\frac{k_{dis}^{CT}}{k_{rec}^{CT}}$. For $\frac{k_{dis}^{CT}}{k_{rec}^{CT}} < 0.5$ a large amount of the photo-generated CT states recombines before generating free charges. This loss can is what is referred to as geminate recombination [18]. The loss in J_{sc} for $\frac{k_{dis}^{CT}}{k_{rec}^{CT}} > 0.5$ is strongly mobility dependent, which suggest that these losses are related to the slow extraction and high non-geminate recombination of free charge carriers. In what follow we will focus on the results where $\frac{k_{dis}^{CT}}{k_{rec}^{CT}} > 0.5$ to only consider cases where the generation of the free charge carrier is reasonably high.

Table S 14 Characteristic of the devices modelled for the CT-drift-diffusion model along with the different parameters explored for the results in figure S15 and figure S16.

Parameter name	Symbol	Hole transfer layer	Intrinsic layer	Electron transfer layer
**Fixed device parameter**				
**Band gap**	E_{CS}	$1.3\ eV$	$1.3\ eV$	$1.3\ eV$
**Built-in voltage**	V_{bi}		$1.1\ V$	
**Dielectric constant**	ε_s	3	3	3
**n-type acceptor density**	N_A	0	0	$2.10^{17}\ cm^{-3}$
**p-type donor density**	N_D	$2.10^{17}\ cm^{-3}$	0	0
**Rate of LE dissociation**	$k_{dis}^{LE,CT}$		$10^{12}\ s^{-1}$	
**Rate of LE recombination**	k_{rec}^{LE}		$10^{10}\ s^{-1}$	
**Thickness**		$300\ nm$	$100\ nm$	$300\ nm$
**Variable device parameter**				
**Electron mobility**	μ_e	$10^{-3}\ cm^2V^{-1}s^{-1}$	$10^{-3}\ to\ 10^{-5}\ cm^2V^{-1}s^{-1}$	$10\ cm^2V^{-1}s^{-1}$
**Hole mobility**	μ_p	$10\ cm^2V^{-1}s^{-1}$	$10^{-3}\ to\ 10^{-5}\ cm^2V^{-1}s^{-1}$	$10^{-3}\ cm^2V^{-1}s^{-1}$
**Free charge to CT state rate constant**	$B_{for}^{CS,CT}$		$10^{-7}\ to\ 10^{-10}\ cm^3s^{-1}$	
**CT state recombination rate constant**	k_{rec}^{CT}		$10^9\ to\ 10^{11}\ s^{-1}$	
**CT state dissociation to free charge rate**	$k_{dis}^{CT,CS}$		$10^9\ to\ 10^{11}\ s^{-1}$	
**Measurement parameters**				
**Generation rate**	G_{av}		$2.5\ 10^{21}\ cm^{-3}s^{-1}$	
**Laser pulse length**		$0.2\ \mu s$	$0.2\ \mu s$	$0.2\ \mu s$
**Laser intensity**		$10^{22}\ cm^{-3}s^{-1}$	$10^{22}\ cm^{-3}s^{-1}$	$10^{22}\ cm^{-3}s^{-1}$
**Offset(E_{s1}-E_{CT})**			0.3 eV	

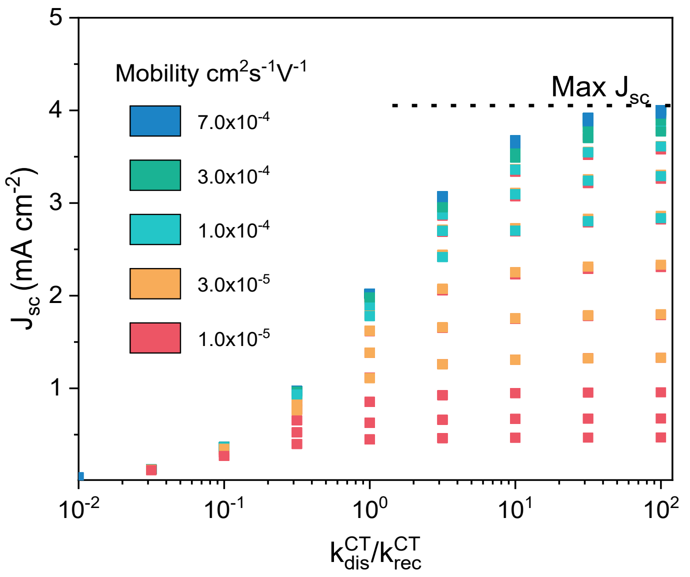

Figure S 15 Results of the CT-Drift-Diffusion model exploration with the parameters in Error! Reference source not found.. **J_{sc} is plotted against the ratio $\frac{k_{rec}^{CT}}{k_{dis}^{CT}}$. The generation rate is 4 times lower than 1 sun conditions considered in the main text.**

In figure S15, we plot the results for the FF, V_{oc}, $\tau_{Q,1}$ and τ_{TPV} against $\frac{k_{rec}^{CT} B_{for}^{CS\backslash CT}}{k_{dis}^{CT}}$, which can be considered as an effective second order recombination rate constant (B^{eff}). We also limit the results to the case where $\frac{k_{dis}^{CT}}{k_{rec}^{CT}} > 0.5$ and FF > 0.4. The FF drops with increased B^{eff} and its drop is mobility dependent. The effect of B^{eff} on the FF is similar to the effect of the bimolecular recombination rate constant in a classical drift diffusion simulation. The correlation between V_{oc} and B^{eff} in figure S16 is comparable to the effect of the bimolecular recombination rate constant in a classical drift diffusion simulation. We find:

$$qV_{oc} = E_{CS} - k_B T \ln\left(\frac{k_{rec}^{CT} B_{for}^{CS\backslash CT} N_{CB} N_{VB}}{k_{dis}^{CT} \quad G_{av}}\right).\#(S14)$$

Since in the simulation we kept N_{CB}, N_{VB} and G_{av} constant, according to equation 10 V_{oc} should be linear with $log(B^{eff})$. First, the change of both the FF and V_{oc} with B^{eff} in figure S16 is analogous to the one expected from the steady state equations: the FF drops with increased B^{eff}, and V_{oc} is linear with the $log(B^{eff})$.

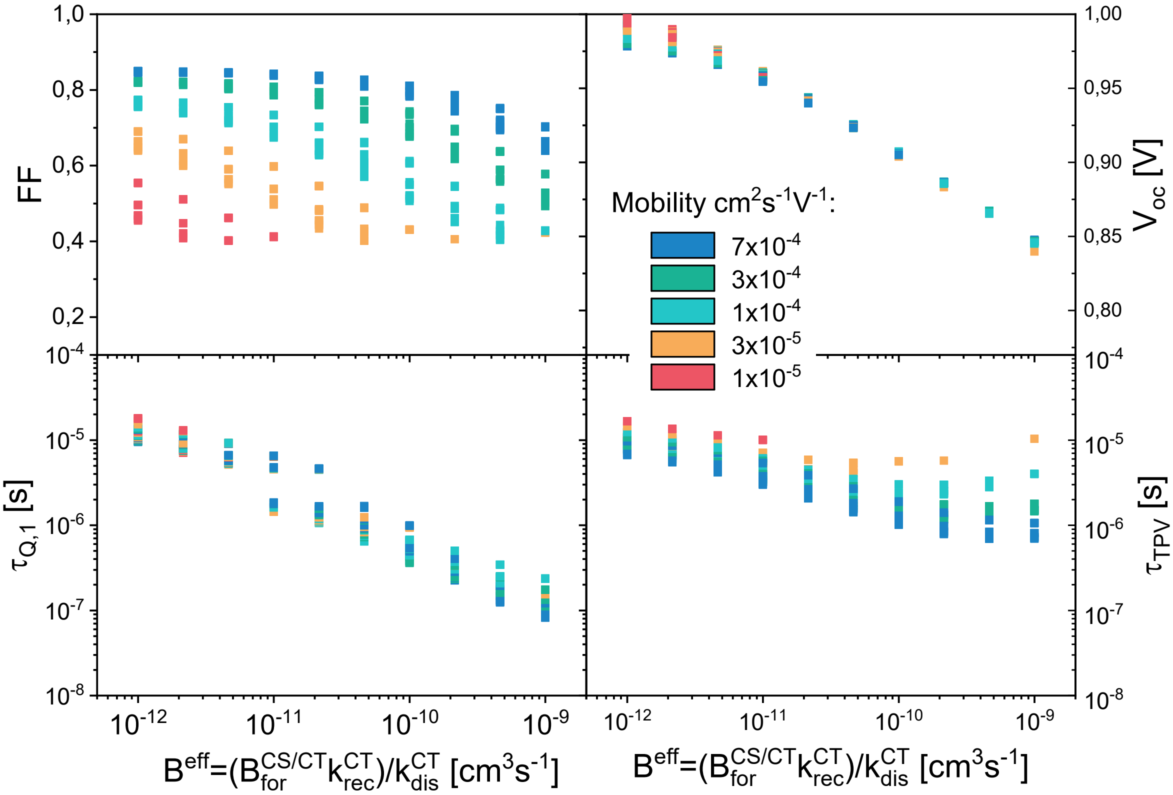

Figure S 16 Results of the CT-Drift-Diffusion model exploration with the parameters in table S14. The characteristics FF, V_{oc}, τ_Q and τ_{TPV}, are plotted against the ratio $\frac{k_{rec}^{CT}B_{for}^{CS\backslash CT}}{k_{dis}^{CT}}$ in figure a to d respectively.

Moving to the change of the time constants $\tau_{Q,1}$ and τ_{TPV} with B^{eff}. For low values of B^{eff} (<10⁻¹¹ cm³s⁻¹), both $\tau_{Q,1}$ and τ_{TPV} decay with increased B^{eff} independently of their mobility. For higher B^{eff}, τ_{TPV} becomes mobility dependent and shows a reversed correlation for low mobility cases, whereas $\tau_{Q,1}$ decay with the same slope as the case for low B^{eff}.

11.2. TPV and TPQ

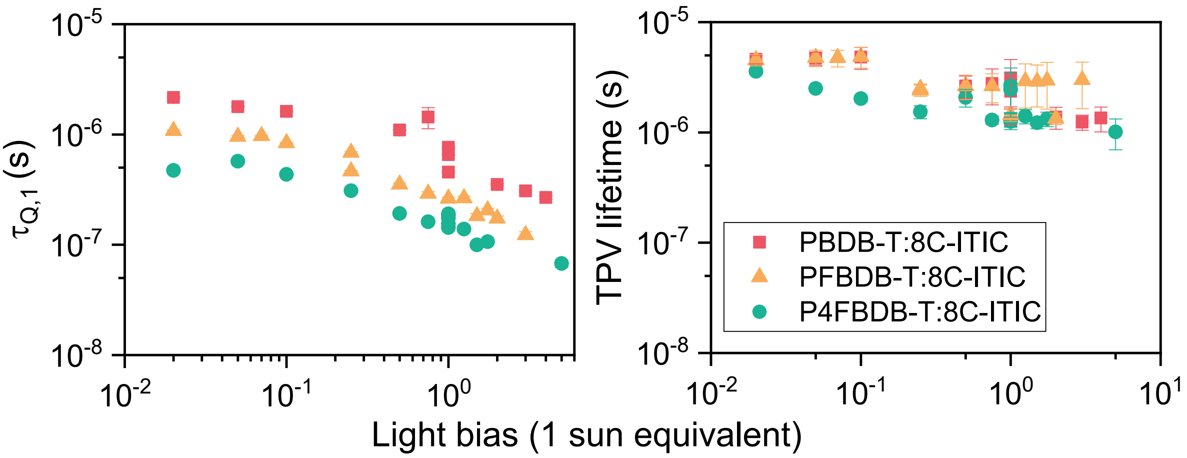

Figure S 17 (a) Lifetime of the free charge carrier estimated using transient photo-charge (TPQ) at different bias light intensities and b) the photovoltage decay time constant (τ_{TPV}) measured using transient photo-voltage (TPV) at different background light intensities.

The measured charge carrier lifetimes measured (τ_Q) using TPQ and the transient photovoltage time constant (τ_{TPV}) for the tree devices is presented in figure S17. The charge carrier lifetime shows a clear decrease at all light intensities with increased fluorination of the donor polymer. On the other hand, the TPV lifetime does not show any clear trend between the three devices. The difference between the two-time constants is due to the limitations of transient photovoltage technique to capture the lifetime of charge carriers in low mobility material [12].

The change of $\tau_Q$with light intensity is similar for the three devices, and show two different regime: 1) a first regime where the time constant is slightly dependent on light intensity and 2) a second regime where the time constant is strongly affected by the light intensity. This suggests that the dominant recombination mechanisms in these devices' changes from a first order process at low light intensity and a second order process for higher light intensity.

11.3. Fixed device parameters:

Table S 15 Fixed device parameters used to simulate the devices in the series

Parameter name	Symbol	Hole transfer layer	Intrinsic layer	Electron transfer layer
**Dielectric constant**	ε_s	3	3	3
**n-type acceptor density**	N_A	0	0	$4.10^{17}\ cm^{-3}$
**p-type donor density**	N_D	$4.10^{17}\ cm^{-3}$	0	0
**Thickness**		$100\ nm$	d	$100\ nm$
**Electron mobility**	μ_e	$10^{-3}\ cm^2V^{-1}s^{-1}$	$3\ 10^{-4}\ cm^2V^{-1}s^{-1}$	$10\ cm^2V^{-1}s^{-1}$
**Hole mobility**	μ_p	$10\ cm^2V^{-1}s^{-1}$	$3\ 10^{-4}\ cm^2V^{-1}s^{-1}$	$10^{-3}\ cm^2V^{-1}s^{-1}$
**Effective DOS in the conduction and valence bands**	N_{CB}, N_{VB}	$2.10^{19}\ cm^{-3}$	$2.10^{19}\ cm^{-3}$	$2.10^{19}\ cm^{-3}$
**Electron affinity**	Φ_{EA}	0	0	**0**

11.4. Key Model parameters changing along the series:

Table S16 Key model parameters that are changing along the series

	Input parameters	PBDB-T	PFBDB-T	P4FBDB-T
Free parameters	$B_{for}^{CS,CT}$ ($cm^3\ s^{-1}$)	$2\ 10^{-11}$	$1.3\ 10^{-10}$	$7.4\ 10^{-10}$
	$k_{dis}^{CT,CS}$ (s^{-1})	$2\ 10^{10}$	$1.6\ 10^{10}$	$4.1\ 10^{9}$
	$k_{dis}^{LE,CT}$ (s^{-1})	$1.7\ 10^{11}$	$2.4\ 10^{11}$	$2.5\ 10^{10}$
	ΔG_{CT}^{0} (eV)	1.31	1.34	1.42
Dependent parameters	$\Delta G_{LE,CT}$(eV)	0.32	0.29	0.21
	E_{CS} (eV)	1.19	1.28	1.44
	$\Delta G_{CT,CS}(eV)$	0.12	0.06	-0.02
	$k_{rec,nrad}^{CT}[s^{-1}]$	$7\ 10^{9}$	$5\ 10^{9}$	$2\ 10^{9}$
	$k_{rec,rad}^{CT}[s^{-1}]$	$3\ 10^{4}$	$4\ 10^{4}$	$5\ 10^{4}$

12. DEVICE PERFORMANCES

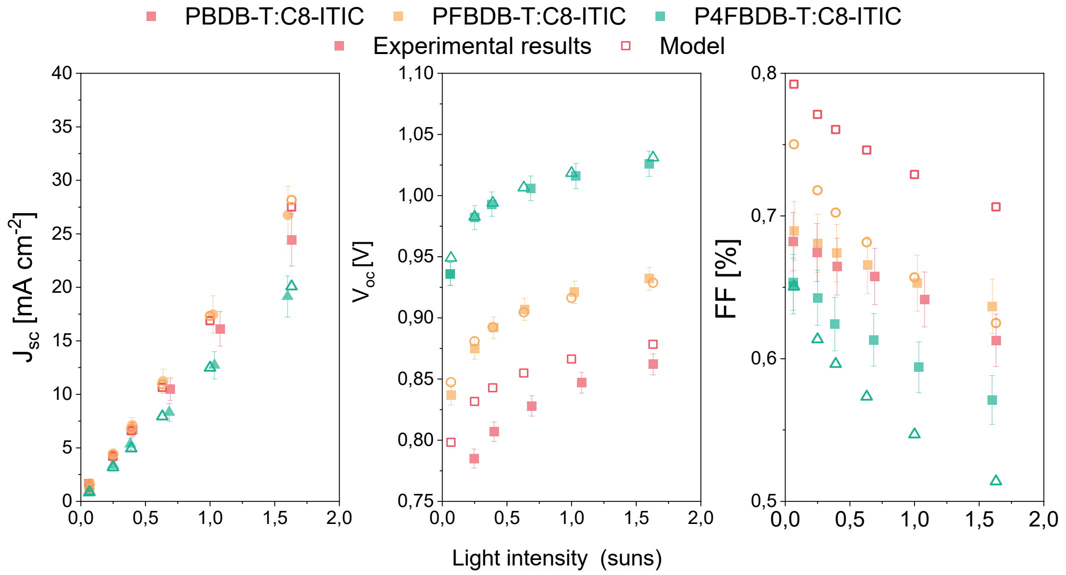

Figure S 18 Summary of measured device performance at different light intensities and the model results.

The measured FF for the PFBDB-T blend is accurately reproduced, but for the PBDB-T blend it is lower than the simulated one and for the P4FBDB-T blend slightly higher than the simulated value. The mismatch between the experimental and simulated FF could be explained by properties of the device that could not be estimated accurately using the experimental measurement used in the study such as: the charge carrier mobilities and the properties of the transport layer impacting the charge carrier injection and extraction properties of the device [16,19].

13. MARCUS RATE FOR THE DISSOCIATION RATES OF EXCITON AND CT STATES.

We can use Marcus rate constant formula to describe the dissociation of the excitons or the dissociation of the CT state [20]. The rate constant can be written as

$$k_M = \frac{2\pi}{\hbar} t_{el}^2 \frac{1}{\sqrt{4\pi\lambda k_B T}} \exp\left(-\frac{(\lambda - \Delta G)^2}{4\lambda k_B T}\right) \quad (S15)$$

Where t_{el} is the electronic coupling between the initial and final state, λ the reorganisation energy of the transition, $\hbar$ the Planck constant, T the temperature and k_B the Boltzmann's constant. ΔG is the difference in free energy between the initial state and the final state. We use this formula to fit the change in the dissociation rates of the LE and the CT state along the series with the electronic coupling and reorganisation energy as fitting parameters. The results of the fit and the model results are presented in table S17.

Table S 17 dissociation rate constant used in the model, and the one calculated using Marcus formula. For the LE to CT dissociation t_{EL}=30 meV and λ=0.54 eV. For the CT to CS dissociation t_{EL}=4 meV and λ=110 meV.

Polymer	$\Delta G_{LE,CT}$ (meV)	Model $k_{dis}^{LE,CT}$ (s-1)	Marcus $k_{dis}^{LE,CT}$ (s-1)	$\Delta G_{CT,CS}$ (meV)	Model $k_{dis}^{CT,CS}$ (s-1)	Marcus $k_{dis}^{CT,CS}$ (s-1)
PBDB-T	320	1.7 10^{11}	2.1 10^{11}	120	2.1 10^{10}	1.9 10^{10}
PFBDB-T	290	2.4 10^{11}	1.7 10^{11}	60	1.6 10^{10}	1.5 10^{10}
P4FBDB-T	210	2.5 10^{10}	7.6 10^{10}	-20	4.1 10^{9}	4.5 10^{9}

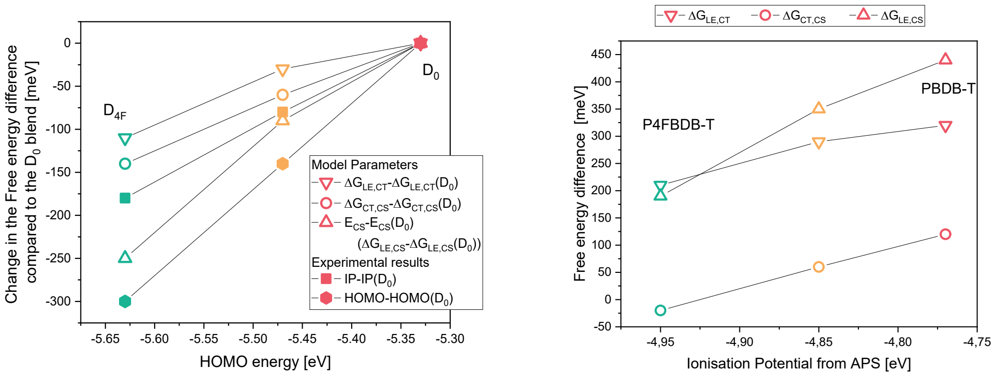

Figure S 19 Comparison of the impact of changing the HOMO energy of the donor on the free energy difference between the excited stated in the model. Ionisation potential of the donor taken from ref [2]. The HOMO energies are estimated from Cyclic voltammetry measurement, and the IP is measured using air photoelectron spectroscopy (APS).

14. REFORMATION RATE CONSTANT AND LANGEVIN ENCOUNTER RATE CONSTANT.

The reformation rate constant of CT state from free charge carrier ($B_{for}^{CS,CT}$) could be approached by the encounter probability in the Langevin recombination framework as has been done by Burke et al. [21]. In this framework $B_{for}^{CS,CT}$ can be approached by $\frac{q(\mu_e+\mu_h)}{\epsilon\epsilon_r}$. We use this formula to back calculate the average charge carrier mobility needed to ensure $B_{for}^{CS,CT}=\frac{q(\mu_e+\mu_h)}{\epsilon\epsilon_r}$ for each of the devices studied (Table S11). Using the mobility extracted from the Langevin model significantly reduces the PCE of the devices as compared to the experimental results. This suggest that the rate of CT reformation cannot be explained by an encounter probability, but the energetics of the interface has an effect on it. From the results of this work, it seems that $B_{for}^{CS,CT}$ increases with reduced energy offset between the HOMO of the donor and the HOMO of the acceptor. $B_{for}^{CS,CT}$ is also found to reduce with reduced $\Delta E_{CT,CS}$.

For the P4FBDB-T blend, $B^{CS,CT}_{for}$ is almost 3 times higher than that estimated using $\frac{q(\mu_e+\mu_h)}{\epsilon\epsilon_r}$. For an average charge carrier mobility of 3 10-4 $cm^2V^{-1}s^{-1}$, $k_L = 2.57\ 10^{-10}\ cm^3s^{-1}$ This means that for this blend with the parameters considered the Langevin reduction factor is >1. Now we discuss the case where the encounter probability is a limiting process (i.e. $B^{CS,CT}_{for} < k_L$). To reproduce the same device properties we need to ensure that $\frac{k^{CT}_{rec}B^{CS\backslash CT}_{for}}{k^{CT}_{dis}}$ is constant (as it controls the lifetime of the free charge carrier), therefore to reduce $B^{CS,CT}_{for}$ we would need to either increase k^{CT}_{rec} or reduce k^{CT}_{dis}. Increasing k^{CT}_{rec} could be achieved by adapting the reorganisation energy of the CT state, however it would increase the voltage losses of the device. On the other hand, reducing k^{CT}_{dis} will change the early time dynamics and reduce the J_{sc} of the device. This means that is it not easy how we can uphold the restriction $B^{CS,CT}_{for} < k_L$, rather we can reconsider the value of the mobility or the dielectric constant considered in this model. Moreover, the enhanced recombination as compared to the Langevin rate constant is not unique to this study and has been related to the disorder and energetic distribution in the blend [22].

Table S 18 Charge carrier mobility estimated using a Langevin encounter probability description for the reformation of CT states from free charge carriers.

Polymer	$\Delta G_{LE,CT}$ (meV)	$\Delta G_{CT,CS}$ (meV)	Model μ ($cm^2V^{-1}s^{-1}$)	Model $B^{CS,CT}_{for}$ ($cm^3\ s^{-1}$)	Langevin μ ($cm^2V^{-1}s^{-1}$)
PBDB-T	**320**	120	$3\ 10^{-4}$	$2\ 10^{-11}$	$2.5\ 10^{-5}$
PFBDB-T	**290**	60	$3\ 10^{-4}$	$1.3\ 10^{-10}$	$1.5\ 10^{-4}$
P4FBDB-T	**210**	-20	$3\ 10^{-4}$	$7.4\ 10^{-10}$	$8.6\ 10^{-4}$

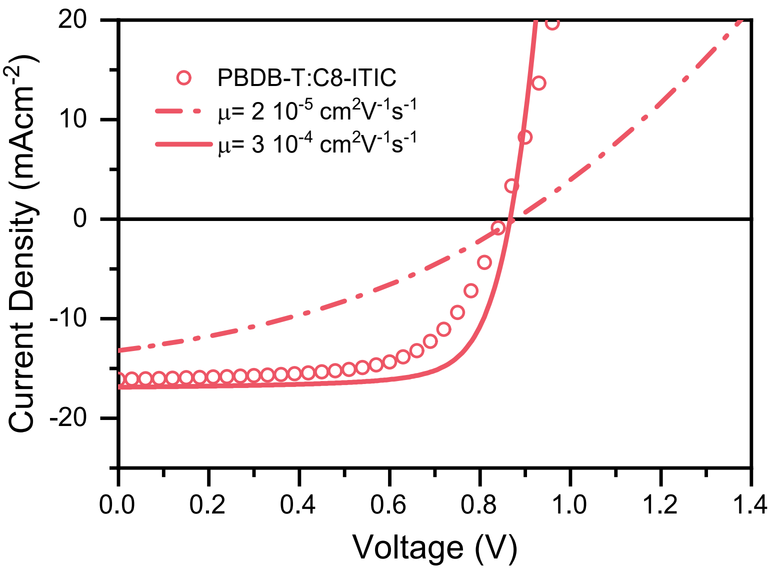

Figure S 20 Modelled JV for the PBDB-T:C8-ITIC blend with the parameters presented in table S6 (LE state properties), table S7 (CT state properties), table S8 (kinetic model rate constants) and table S15 (device properties). The measured experimental result is presented with open circles. The Two model results consider either the charge carrier mobility considered in this work (3 10-4 $cm^2V^{-1}s^{-1}$), and the one estimated in the Langevin recombination framework for this device (2.5 10-5 $cm^2V^{-1}s^{-1}$).

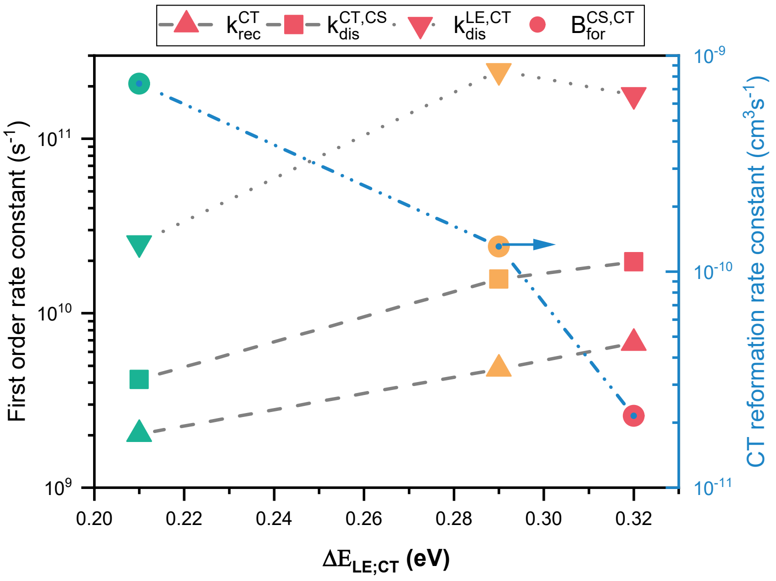

Figure S 21 plot summarising the free parameters considered to reproduce the three blend considered in the case study and their dependence on the energy offset between the CT and LE state.

15. IMPROVING THE EFFICIENCY OF LOW OFFSET BLENDS.

We have found that reducing the offset between the HOMO of the donor and the HOMO of the acceptor affects different processes in the devices (dissociation of the CT and LE state and the reformation of the CT state from free charges). To improve the efficiency of the lowest offset system we propose to increase the CT state dissociation rate constant ($k^{CT,CS}_{dis}$). This could be achieved by increasing the electronic coupling between the charge separated and the CT state or a reduced reorganisation energy between the two states. When increasing $k^{CT,CS}_{dis}$, we need to ensure that $[CT]_0 k^{CT}_{dis} = B^{CS,CT}_{for} n_i^2$, to ensure that the equilibrium is respected. Therefore, we can either increase both k^{CT}_{dis} and $B^{CS,CT}_{for}$ (case 1) , or increasing k^{CT}_{dis} and n_i (we can increase n_i by increasing $N_{CB} and\ N_{VB}$) (case 2).

15.1. Case 1 : increasing both k^{CT}_{dis} and $B^{CS,CT}_{for}$.

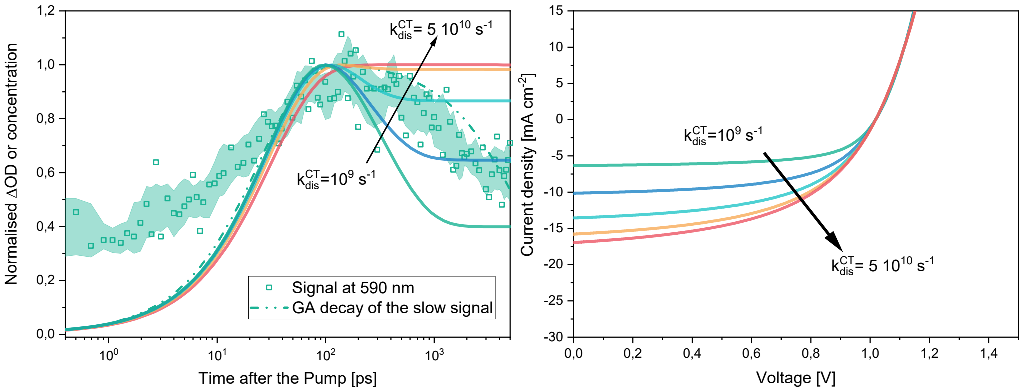

Figure S 22 GSB dynamics of the modelled P4FBDB-T blend with different CT state dissociation rate constant. B) modelled JV of the P4BDB-T:C8-ITIC blend with different dissociation rate constants of the CT states.

In this first case, when increasing k^{CT}_{dis} we improve the charge generation efficiency. Since $B^{CS,CT}_{for}$ is increased simultaneously the device performance does not increase significantly due to a reduced FF. Table S15 summarises these results.

Table S 19 Device performances under 1 sun illumination, for the case where the dissociation rate constant and the reformation rate constants are changed simultaneously. The rest of the device parameters are the same for the P4FBDB-T:8C-ITIC in the main paper.

$k^{CT,CS}_{dis}$ (s^{-1})	$B^{CS,CT}_{for}$ (cm^3 s^{-1})	J_{sc} (mA cm^{-2})	V_{oc} (V)	FF (%)	PCE (%
**10^9**	10^{-10}	6.33	1.02	0.64	4.11
**$2\ 10^9$**	$4\ 10^{-10}$	10.15	1.02	0.58	5.96
**$5\ 10^9$**	10^{-9}	13.56	1.02	0.53	7.38
**10^{10}**	$2\ 10^{-9}$	15.79	1.02	0.51	8.21
**$3\ 10^{10}$**	$5\ 10^{-9}$	16.95	1.02	0.50	8.62
**$5\ 10^{10}$**	$8\ 10^{-9}$	17.51	1.03	0.50	9.00
**10^{11}**	$2\ 10^{-8}$	17.86	1.03	0.50	9.12

15.2. Case 2 : increasing both k_{dis}^{CT} and n_i

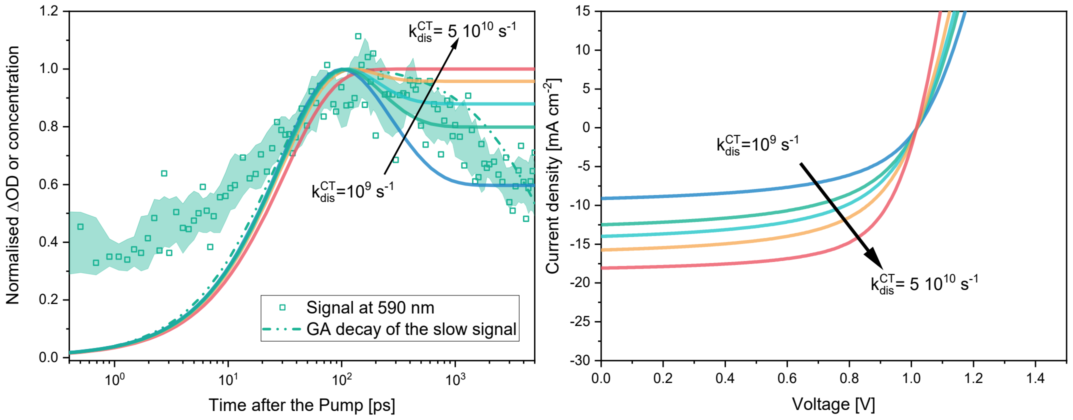

Figure S 23 GSB dynamics of the modelled P4FBDB-T blend with different CT state dissociation rate constant. B) modelled JV of the P4BDB-T:C8-ITIC blend with different dissociation rate constants of the CT states.

In this case we increase the rate of dissociation $k_{dis}^{CT,CS}$ and simultaneously increase N_{CB},N_{VB}. Since the reformation rate constant is kept constant, the FF of the devices is increased with increased dissociation rate of the CT state. This is because the FF is affected by the ratio ($\frac{k_{rec}^{CT} B_{for}^{CS\backslash CT}}{k_{dis}^{CT;CS}}$). In this case the device can reach up to 13.4 % PCE, due to both an increase in J_{sc} and the FF.

Table S 20 Device performances under 1 sun illumination, for the case where the dissociation rate constant and N_{CB},N_{VB} are changed simultaneously. The rest of the device parameters are the same for the P4FBDB-T:8C-ITIC in the main paper.

$k_{dis}^{CT,CS}$ (s^{-1})	$B_{for}^{CS,CT}$ (cm^3 s^{-1})	N_{CB},N_{VB} (cm^{-3})	J_{sc} (mA cm^{-2})	V_{oc} (V)	FF (%)	PCE (%
**10^9**	7 10^{-10}	10^{19}	6.45	1.02	0.54	3.55
**2 10^9**	7 10^{-10}	1.4 10^{19}	10.16	1.03	0.54	5.58
**5 10^9**	7 10^{-10}	2.2 10^{19}	13.75	1.03	0.56	7.85
**10^{10}**	7 10^{-10}	3.3 10^{19}	16.42	1.03	0.60	10.04
**3 10^{10}**	7 10^{-10}	5 10^{19}	18.04	1.03	0.65	11.94
**6 10^{10}**	7 10^{-10}	7.6 10^{19}	18.91	1.02	0.69	13.44

16. CASE WHERE THE CT TO CS STATE TRANSITION IS INDEPENDENT OF THE ENERGY OFFSET.

In this study we have found that changing the HOMO or the ionisation potential of the donor not only affects the LE to CT electron transfer process ($k_{dis}^{LE,CT}$) but also the transition from the CT to CS (through both $k_{dis}^{CT,CS}$ and $B_{for}^{CS,CT}$). In this section, we explore the case where

we consider that changing the energetics of the donor impact both the CT and CS states energies in a similar way, so that $\Delta G_{CT,CS} \approx \Delta G^{0}_{CT} - E_{CS}$ is constant. This assumption means that the rate of CT dissociation and reformation are the same for all the blends.

For this case we consider that the $\Delta G_{CT,CS}$, $k^{CT,CS}_{dis}$ and $B^{CS,CT}_{for}$ are the same for all the devices as the value estimated for the PBDB-T blend. Then the impact of the fluorination of the donor on the CS state energy (E_{CS}) is kept the same as the one inferred from the model above. This assumption and their impact on the different parameters of the model are summarised in table S21. The other device parameters are kept the same as in the main model used in the paper. Here we refer to the main model it as the four free parameter model (or case 1), the model with fixed CT to CS state transition as the model with fixed $\Delta G_{CT,CS}$ (or case 2). The rate of exciton dissociation ($k^{LE,CT}_{dis}$) is calculated in this fixed $\Delta G_{CT,CS}$ case using the results of section 13 (using equation S15 and t_{EL}=30 meV and λ=0.54 eV).

Table S 21 Parameter consider for the case where the CT to CS state transition is independent of the energy offset.

		PBDB-T	PFBDB-T	P4FBDB-T
Fixed parameters	$B^{CS,CT}_{for}$ (cm^3 s^{-1})	2 10^{-11}	2 10^{-11}	2 10^{-11}
	$k^{CT,CS}_{dis}$ (s^{-1})	2 10^{10}	2 10^{10}	2 10^{10}
	$\Delta G_{CT,CS}(eV)$	0.12	0.12	0.12
Free parameter	E_{CS} (eV)	1.19	1.28	1.44
Dependent parameters	$k^{LE,CT}_{dis}$ (s^{-1})	1.7 10^{11}	2.4 10^{11}	2.5 10^{10}
	ΔG^{0}_{CT} (eV)	1.31	1.39	1.55
	$\Delta G_{LE,CT}$(eV)	0.32	0.24	0.08
	$k^{CT}_{rec,nrad}[s^{-1}]$	7 10^9	2.5 10^9	7.3 10^8
	$k^{CT}_{rec,rad}[s^{-1}]$	3.5 10^4	4.8 10^4	1.7 10^5

The comparison of the GSB dynamics simulated with the two sets of parameters (figure S24). show that the model with fixed $\Delta G_{CT,CS}$ has a slower rise for both PFBDB-T and P4FBDB-T blends. Moreover, due to the low recombination rate constant of the CT state ($k_{rec,nrad}^{CT}$), the GSB does shows a small drop as compared to the model with the 4 free parameters. This shows that for we need to consider a change in the CT to CS transition to accurately reproduce the experimental results.

When comparing the simulated luminescence and EQE spectra of the two sets of parameters (figure S25), we find that the EL and EQE spectra for case 2 is blue shifted as compared to both the experimental results and the spectra from case 1.

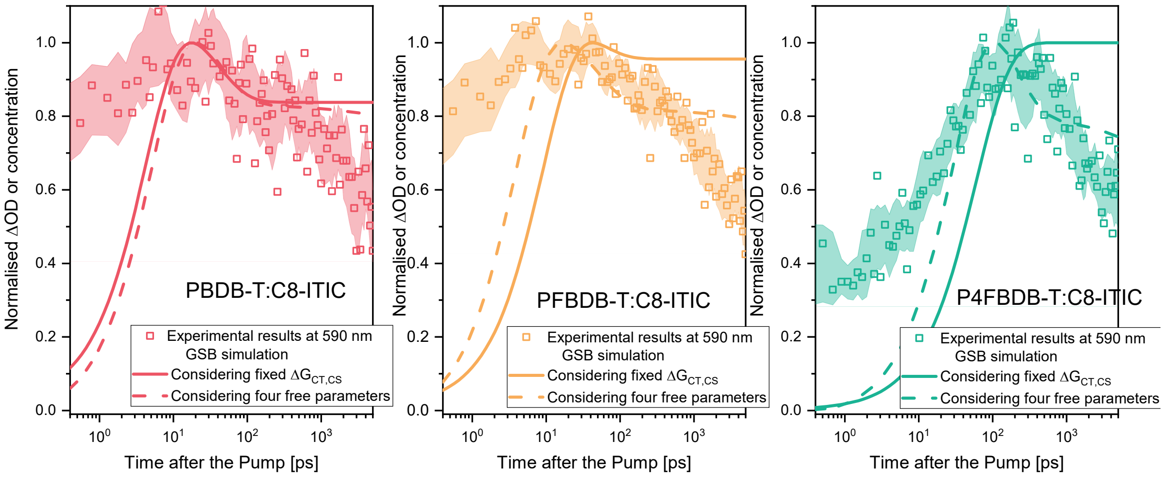

Figure S 24 GSB dynamics simulated using he two models compared the experimental results probed at 590 nm.

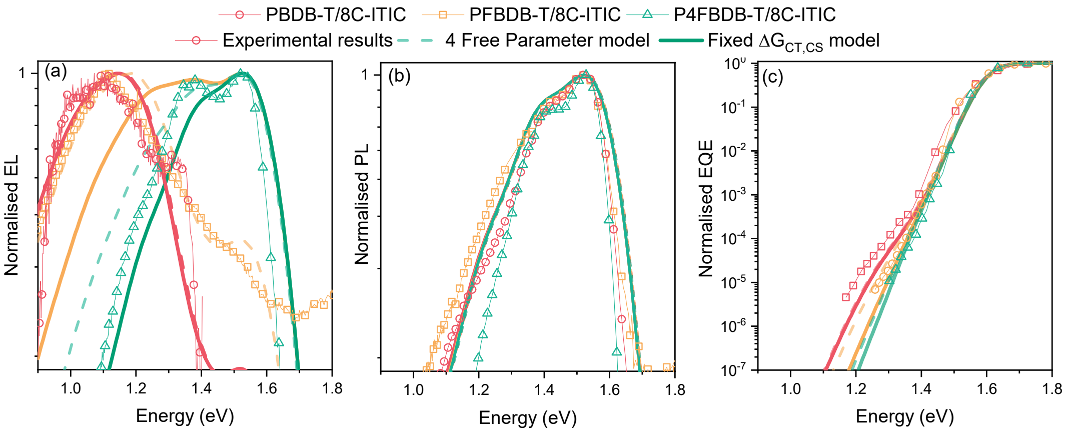

Figure S 25 Modelled and experimental EL, PL and EQE.

Finally for the device characteristics of the device, we find that if the CT to CS energy offset was kept the same (case 2) the lowest offset device would have a considerably higher PCE, mainly due to the strong increase in the V_{oc}, without affecting the FF.

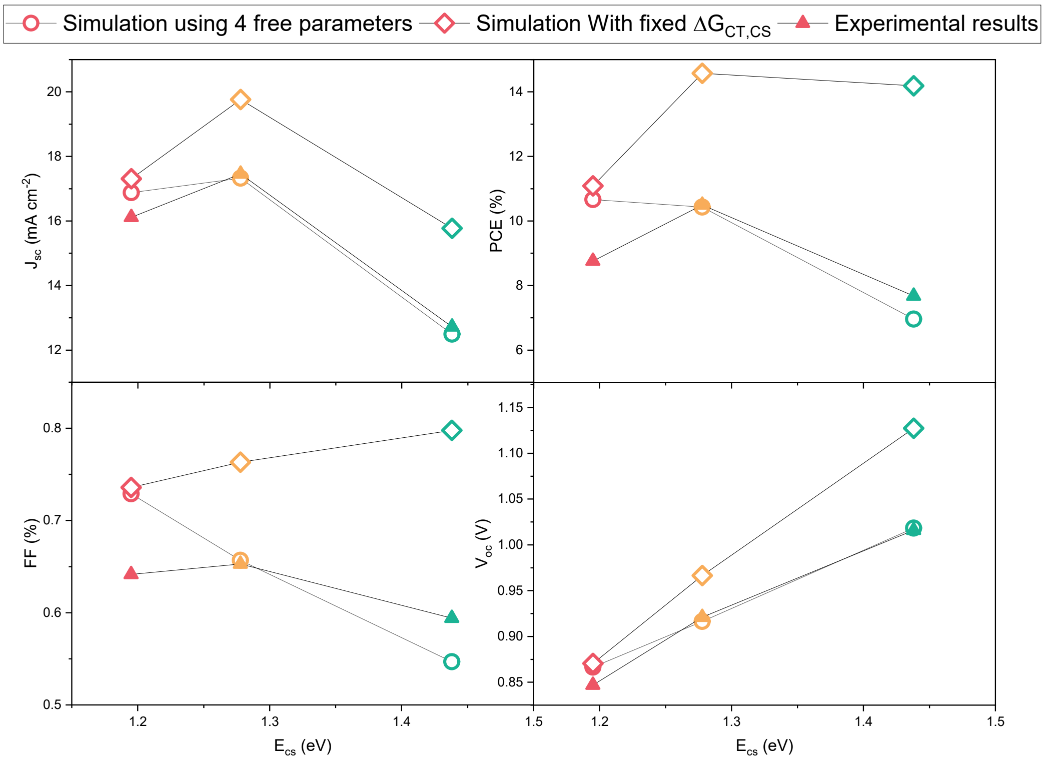

Figure S 26 Experimental device characteristics at AM1.5 simulated light intensity compared to the model results using the two sets of parameters.

17. REFERENCES

1 Z. Fei, F. D. Eisner, X. Jiao, M. Azzouzi, J. A. Röhr, Y. Han, M. Shahid, A. S. R. Chesman, C. D. Easton, C. R. McNeill, T. D. Anthopoulos, J. Nelson and M. Heeney, *Advanced Materials*, 2018, **30**, 1705209.

2 F. D. Eisner, M. Azzouzi, Z. Fei, X. Hou, T. D. Anthopoulos, T. J. S. Dennis, M. Heeney and J. Nelson, *Journal of the American Chemical Society*, 2019, **141**, 6362–6374.

3 P. Calado, A. M. Telford, D. Bryant, X. Li, J. Nelson, B. C. O'Regan and P. R. F. Barnes, *Nature Communications*, 2016, **7**, 13831.

4 B. Blank, T. Kirchartz, S. Lany and U. Rau, *Physical Review Applied*, 2017, **8**, 024032.

5 T. Kirchartz, *Generalized detailed balance theory of solar cells*, Forschungszentrum Jülich, 2009, vol. 38.

6 A. Classen, C. L. Chochos, L. Lüer, V. G. Gregoriou, J. Wortmann, A. Osvet, K. Forberich, I. McCulloch, T. Heumüller and C. J. Brabec, *Nature Energy*, 2020, **5**, 711–719.

7 M. C. Heiber, C. Baumbach, V. Dyakonov and C. Deibel, *Physical Review Letters*, 2015, 3–5.

8 J. Yao, T. Kirchartz, M. S. Vezie, M. A. Faist, W. Gong, Z. He, H. Wu, J. Troughton, T. Watson, D. Bryant and J. Nelson, *Physical Review Applied*, 2015, **4**, 014020.

9 M. Azzouzi, J. Yan, T. Kirchartz, K. Liu, J. Wang, H. Wu and J. Nelson, *Physical Review X*, 2018, **8**, 031055.

10 S. Few, J. M. Frost, J. Kirkpatrick and J. Nelson, *The Journal of Physical Chemistry C*, 2014, **118**, 8253–8261.

11 L. J. G. W. van Wilderen, C. N. Lincoln and J. J. van Thor, *PLoS ONE*, 2011, **6**, 17373.

12 M. Azzouzi, P. Calado, A. M. Telford, F. Eisner, X. Hou, T. Kirchartz, P. R. F. Barnes and J. Nelson, *Solar RRL*, 2020, **4**, 1900581.

13 J. A. Röhr, X. Shi, S. A. Haque, T. Kirchartz and J. Nelson, *Physical Review Applied*, 2018, **9**, 44017.

14 H. K. H. Lee, A. M. Telford, J. A. Röhr, M. F. Wyatt, B. Rice, J. Wu, A. de Castro Maciel, S. M. Tuladhar, E. Speller, J. McGettrick, J. R. Searle, S. Pont, T. Watson, T. Kirchartz, J. R. Durrant, W. C. Tsoi, J. Nelson and Z. Li, *Energy & Environmental Science*, 2018, **11**, 417–428.

15 R. C. I. MacKenzie, T. Kirchartz, G. F. A. Dibb and J. Nelson, *The Journal of Physical Chemistry C*, 2011, **115**, 9806–9813.

16 B. Xiao, P. Calado, R. C. I. MacKenzie, T. Kirchartz, J. Yan and J. Nelson, *Physical Review Applied*, 2020, **14**, 024034.

17 R. C. I. MacKenzie, *https://www.gpvdm.com.*

18 D. Credgington, F. C. ; Jamieson, B. Walker, T.-Q. Nguyen and J. R. Durrant, *Advanced Materials*, 2012, **24**, 2135–2141.

19 C. M. Proctor and T. Q. Nguyen, *Applied Physics Letters*, 2015, **106**, 083301.

20 D. Qian, Z. Zheng, H. Yao, W. Tress, T. R. Hopper, S. Chen, S. Li, J. Liu, S. Chen, J. Zhang, X.-K. Liu, B. Gao, L. Ouyang, Y. Jin, G. Pozina, I. A. Buyanova, W. M. Chen, O. Inganäs, V. Coropceanu, J.-L. Bredas, H. Yan, J. Hou, F. Zhang, A. A. Bakulin and F. Gao, *Nature Materials*, 2018, **17**, 703–709.

21 T. M. Burke, S. Sweetnam, K. Vandewal and M. D. McGehee, *Advanced Energy Materials*, 2015, **5**, 1500123.

22 S. V. Novikov, *Journal of Physical Chemistry C*, 2019, **123**, 18854–18860.
